# Integrated eQTL mapping approach reveals genomic regions regulating candidate genes of the *E8-r3* locus in soybean

**DOI:** 10.3389/fpls.2024.1463300

**Published:** 2024-11-12

**Authors:** Jérôme Gélinas Bélanger, Tanya Rose Copley, Valerio Hoyos-Villegas, Louise O’Donoughue

**Affiliations:** ^1^ Soybean Breeding and Genetics Lab, Centre de recherche sur les grains (CÉROM) Inc., St-Mathieu-de-Beloeil, QC, Canada; ^2^ Department of Plant Science, McGill University, Montréal, QC, Canada

**Keywords:** expression quantitative trait loci, regulatory hotspots, early-maturing soybeans, candidate genes, transcription factor, E8-r3 locus, co-expression network analysis

## Abstract

Deciphering the gene regulatory networks of critical quantitative trait loci associated with early maturity provides information for breeders to unlock soybean’s (*Glycine max* (L.) Merr.) northern potential and expand its cultivation range. The *E8-r3* locus is a genomic region regulating the number of days to maturity under constant short-day photoperiodic conditions in two early-maturing soybean populations (QS15524_F2:F3_ and QS15544_RIL_) belonging to maturity groups MG00 and MG000. In this study, we developed a combinatorial expression quantitative trait loci mapping approach using three algorithms (ICIM, IM, and GCIM) to identify the regions that regulate three candidate genes of the *E8-r3* locus (*Glyma.04G167900*/*GmLHCA4a*, *Glyma.04G166300*/*GmPRR1a*, and *Glyma.04G159300*/*GmMDE04*). Using this approach, a total of 2,218 *trans* (2,061 genes)/7 *cis* (7 genes) and 4,073 *trans* (2,842 genes)/3,083 *cis* (2,418 genes) interactions were mapped in the QS15524_F2:F3_ and QS15544_RIL_ populations, respectively. From these interactions, we successfully identified two hotspots (F2_GM15:49,385,092-49,442,237 and F2_GM18:1,434,182-1,935,386) and three minor regions (RIL_GM04:17,227,512-20,251,662, RIL_GM04:31,408,946-31,525,671 and RIL_GM13:37,289,785-38,620,690) regulating the candidate genes of *E8-r3* and several of their homologs. Based on co-expression network and single nucleotide variant analyses, we identified *ALTERED PHLOEM DEVELOPMENT* (*Glyma.15G263700*) and *DOMAIN-CONTAINING PROTEIN 21* (*Glyma.18G025600*) as the best candidates for the F2_GM15:49,385,092-49,442,237 and F2_GM18:1,434,182-1,935,386 hotspots. These findings demonstrate that a few key regions are involved in the regulation of the E8-r3 candidates *GmLHCA4a*, *GmPRR1a*, and *GmMDE04*.

## Introduction

Soybean (*Glycine max* (L.) Merr.) is the most important leguminous oilseed crop and significantly contributes to maintaining food security on a global scale. This crop is mainly cultivated in countries located in warm temperate, subtropical, and/or tropical areas, and the contribution of northern countries such as Canada (2%) to the global soybean output remains modest (The American Soybean Association, 2023). Current projections suggest limited growth in soybean production for countries located in tropical and subtropical countries (Ali et al., 2022); however, due to the projected rise in world population and anticipated growth in the international need for soybean-related food and industrial goods, global production will need to increase to supply the growing demand (Unc et al., 2021). One approach to partly solve this problem is to improve soybean adaptability to northern regions beyond its actual limits (~54°N) and fine-tune its reproductive phenology by identifying the critical transcription factors regulating the extra-early flowering and maturity phenotypes.

Transcription factors (TFs) are critical proteins that regulate the transcription of one or multiple downstream targets by binding to *cis*-regulatory elements (CRE) with their DNA-binding domains (DBD) (Bylino et al., 2020). In soybean, 6,150 TFs (3,747 loci) belonging to 57 families have been predicted (Jin et al., 2017), with several having reported flowering regulatory functions such as *E1* (Xia et al., 2012), *E1-like-a* (Liu et al., 2022), *E1-like-b* (Zhu et al., 2019), and *LHY1a/1b/2a/2b* (*Glyma.16G017400*, *Glyma.07G048500*, *Glyma.19G260900*, and *Glyma.03G261800*, respectively) (Bian et al., 2017). In *Arabidopsis*, core regulators of the circadian clock are all TFs and include *CIRCADIAN CLOCK-ASSOCIATED1* (*AtCCA1*), *LATE ELONGATED HYPOCOTYL* (*AtLHY*), and the evening-expressed gene, *TIMING OF CAB EXPRESSION1* (*AtTOC1*) (Wang and Ma, 2013). As the main biological timekeeper, the circadian clock gates the global molecular response to the environmental cues, the zeitgebers, in a timely fashion. From an agronomical standpoint, these multiple interlocked transcription-translation feedback loops comprised within the circadian clock regulate essential metabolic functions (e.g. photosynthesis and reproductive phenology) with potential effects on critical traits such as maturity, yield, and disease resistance (Hotta, 2021). In particular, the cryptochrome and phytochrome photoreceptors regulate many key aspects of the circadian clock and act as a molecular bridge between photosynthesis, development, and reproduction (Venkat and Muneer, 2022). As a consequence, photosynthesis and reproduction are intertwined at the molecular level due to specific genes (e.g. *PHYTOCHROME A2/A3*) acting to control photoperiodic flowering (Lin et al., 2022).

Generating a compendium of interactions for one specific TF is challenging due to the transient nature of the regulatory mechanisms and the intricate density of the underlying regulatory networks. One approach to solve this issue is to perform expression quantitative trait loci (eQTL) mapping on a genome-wide scale to identify proximal/*cis* (within a 1-Mbp window of the transcription start site) and distal/*trans* single nucleotide polymorphisms influencing the level of messenger RNA (mRNA) expression (Gilad et al., 2008; Westra and Franke, 2014). Expression quantitative trait loci hotspots are genetic variations, most often located in genes coding for TFs, that regulate the expression level of numerous genes, often in the hundreds to thousands (Choi et al., 2020). Obtaining sufficient statistical power is often challenging in eQTL mapping due to the prohibitive financial cost associated with sufficient reading depth and the computational burden of transcriptome-wide measurements on hundreds of lines. To overcome this challenge, numerous mapping algorithms, such as Genome-wide composite interval mapping (GCIM) (Zhang et al., 2020) and Inclusive Composite Interval mapping (Li et al., 2007), have been developed to improve the identification of small-effect eQTLs, which are most often located in *trans* (Westra and Franke, 2014). We believe that used in conjunction, these methods have an increased ability to identify regions of interest for given phenotypes and can also be used to map eQTL interactions and associated regulatory hotspots with increased precision.

In a previous study, we identified a QTL region named *E8-r3* located between the GM04:41,808,599 and GM04:42,376,237 flanking markers that regulates the number of days to maturity under a constant short-day photoperiod in two early-maturing soybean populations (QS15524_F2:F3_ and QS15544_RIL_) (Gélinas Bélanger et al., 2024a). The same region was not identified when these populations were grown under fluctuating long-day conditions under Canadian field conditions, suggesting that this region is specifically involved in photoperiodic responses under short days. In this previous study, we also identified that this region regulates the expression of several genes, including *E6* (*Glyma.04G050200*), an ortholog of *Arabidopsis thaliana EARLY FLOWERING 3* that has been demonstrated to have an effect on the flowering of soybean using a combinatorial eQTL mapping approach (Fang et al., 2021; Gélinas Bélanger et al., 2024a). The associated QTL region identified for the short-day phenotypic response encompasses 29 genes and is implicated in the ‘Photosynthesis - antenna proteins’ KEGG pathway. In total, we have proposed three candidate genes (*Glyma.04G168300*, *Glyma.04G167900*, and *Glyma.04G166300*) for this region based on a candidate single nucleotide polymorphisms (SNPs) analysis. Two of these genes, *Glyma.04G166300* (*PSEUDO–RESPONSE REGULATOR 1a*; *GmPRR1a*) (Liu et al., 2009; Dietz et al., 2022) and *Glyma.04G168300* (*CYCLING DOF FACTOR 3*; *GmCDF3*) (Corrales et al., 2017), encode TFs involved in the circadian clock, developmental processes and regulation of maturity, thus suggesting that TFs might be involved in the regulation of the *E8-r3* locus. The other gene, *Glyma.04G167900* (*LIGHT-HARVESTING CHLOROPHYLL-PROTEIN COMPLEX I SUBUNIT A4a; LHCA4a*), is involved in photosynthetic activities and possibly regulated by TFs located in *cis* or in *trans*. Recently, a gene coding for a MADS-box transcription factor, *Glyma.04G159300* (*MADS-BOX DOWNREGULATED BY E1 04*; *GmMDE04*), was found to be statistically associated with the GM04:39,294,836 marker for the flowering time (i.e. R1 stage), maturity (i.e. R8 stage), and reproductive length (i.e. the difference between R8 and R1) traits (Escamilla et al., 2024). Although this gene is located outside of *E8-r3* flanking markers, our lab is currently reconsidering its potential role as a regulator for this locus based on the results found by Escamilla et al. (2024). The objective of the present study is to identify novel eQTLs using an approach combining multiple mapping techniques in two early-maturing soybean populations. Overall, this study aims at (i) validating an eQTL mapping pipeline based on a combinatorial mapping strategy; (ii) identifying eQTL signals and hotspots regulating the genes involved in flowering, maturity, and photosynthesis; (iii) locating the eQTL signals interacting with the *E8-r3* region; and (iv) identifying candidate TFs and characterizing their co-expression networks.

## Materials and methods

### Plant materials, growing conditions and phenotyping

The populations and phenotyping procedures were generated and performed as detailed in Gélinas Bélanger et al. (2024a). Briefly, the QS15524_F2:F3_ population was generated from a biparental cross between ‘Maple Arrow’ (MG00; later-maturing accession) × ‘OAC Vision’ (PI 567787) (MG000; earlier-maturing accession), now herein respectively referred as MA and OV. The QS15544_RIL_ population was generated from the biparental cross between ‘AAC Mandor’ (MG00; later-maturing accession) × ‘9004’ (MG000; earlier-maturing accession), the former now being herein referred to as MD. The parental lines in each population were fixed for their *E1* (*Glyma.06G207800*) (Xia et al., 2012), *E2* (*Glyma.10G221500*) (Watanabe et al., 2011), *E3* (*Glyma.19G224200*) (Watanabe et al., 2009), and *E4* (*Glyma.20G090000*) (Liu et al., 2008) alleles. As such, the genotypes for the QS15524_F2:F3_ parental lines were *e1-nl*/*e2-ns*/*E3Ha*/*e4-SORE-1* and *e1-as*/*e2-ns*/*e3-tr*/*e4p.T832QfsX21* for the QS15544_RIL_ parental lines. The *e4p.T832QfsX21* allele is a rare premature stop codon mutation previously identified in Tardivel et al. (2019).

The QS15524_F2:F3_ population was grown and phenotyped in a greenhouse during the winter of 2017-2018 at the Centre de recherche sur les grains inc. (CÉROM) in St-Mathieu-de-Beloeil (QC, Canada), GPS coordinates 45°34’57.9”N 73°14’11.4”W. In the case of QS15544_RIL_, the population (F_5_:F_6_ generation) was grown and phenotyped in a greenhouse during the winter of 2019-2020. Plants for the offspring and parental lines were sown on December 14^th^ 2017 and October 25^th^ 2019 for the QS15524_F2:F3_ and QS15544_RIL_ populations, respectively. During the experiments, natural photoperiod was below 12h but maintained artificially at 12h using sodium halogen lights at all time before flowering since flowering for all plants happened before the March Equinox. Both populations were grown following a custom Modified Augmented Design (Lin & Poushinsky, 1983, 1985) with 19 individuals per table and one parent per table. For each population, the plants were sown in one-gallon pots containing a ProMix-garden soil (1:1 v:v) (Premier Tech Horticulture, Rivière-du-Loup, QC, Canada) potting mix. For the QS15524_F2:F3_ offspring, one seed was planted per pot, whereas three seeds were sown per pot for the QS15544_RIL_ offspring. As reported by Gélinas Bélanger et al. (2024a), the OV and MA parents of the QS15524_F2:F3_ population respectively matured in 85 and 96 days. For the QS15544_RIL_ population, it was observed that the MD and ‘9004’ lines matured in 87.5 and 87.2 days, respectively.

### Sampling, nucleic acid extraction and sequencing

**Figure 1:**
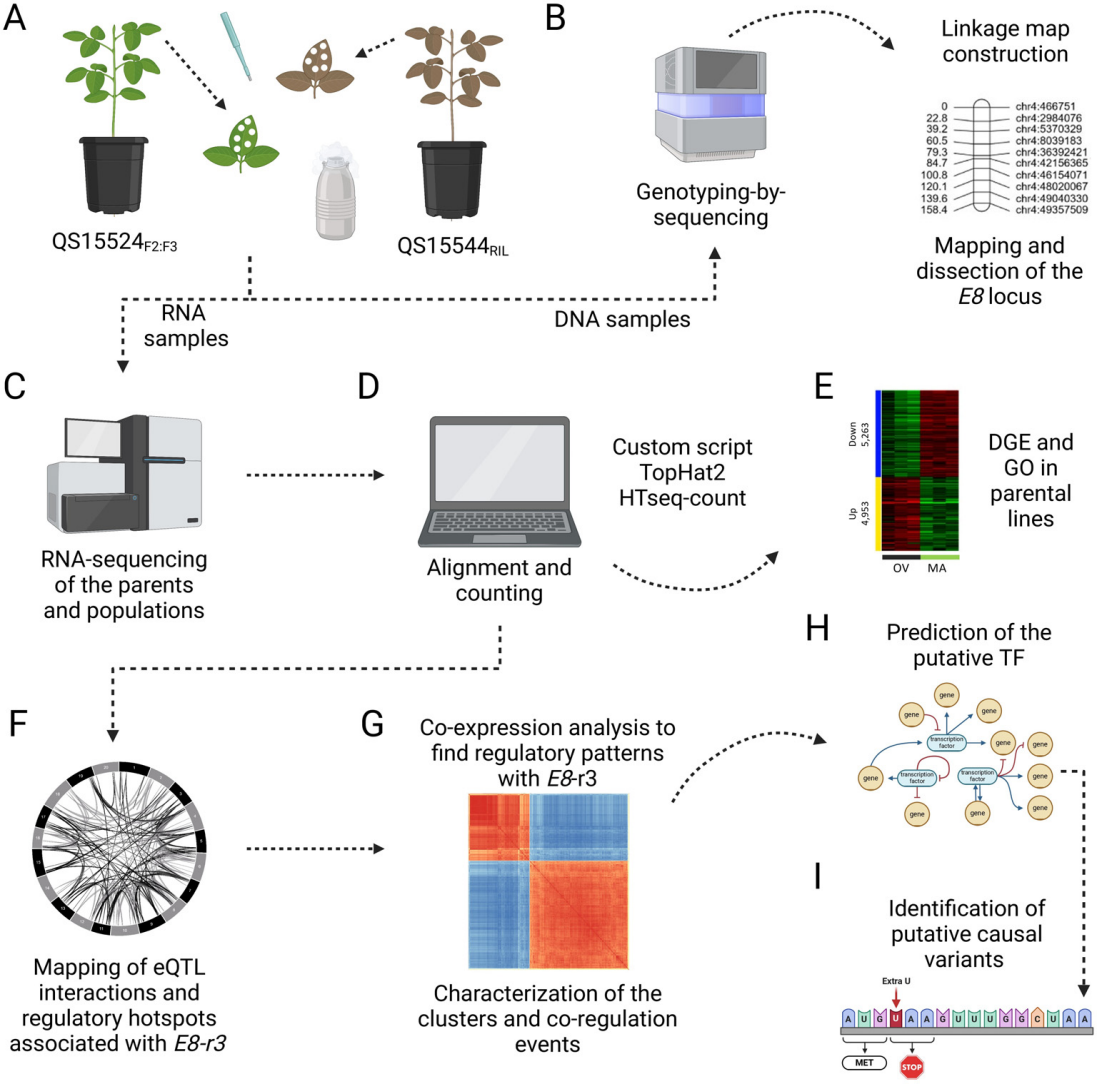
Experimental pipeline to identify candidate TFs involved in the regulation of *E8-r3* genes. **(A)** Leaf tissue collection of the middle leaflet at the V4 stage followed by DNA and RNA extraction. **(B)** Generation of the linkage map with the genotyping-by-sequencing datasets and mapping of the *E8-r3* candidate genes with ICIM and GCIM as detailed in Gélinas Bélanger et al. (2024a). **(C)** Isolation of mRNA and sequencing on the Illumina NovaSeq6000 platform. **(D)** Alignment and counting of the RNA-seq datasets using various bioinformatic scripts. **(E)** Identification of differentially expressed genes in the parental lines to validate the experimental conditions. **(F)** Mapping of the overlapping eQTL interactions using three algorithms (ICIM, IM, and GCIM) and identification of the *trans* interactions associated with *GmLHCA4a*, *GmPRR1a*, and *GmMDE04* candidate genes found in the *E8-r3* region. **(G)** Building of CENs between the genes to identify homologous genes and closely associated candidate TFs. **(H)** Deeper investigations of the candidate TFs using TWCENs, gene expression, and FRSPD_GO annotations. **(I)** Identification of putative causal variants in the candidate TFs. Created with BioRender.com.

The sampling and sequencing procedures were performed as detailed in Gélinas Bélanger et al. (2024a). Briefly, leaf tissue for RNA extraction was harvested by making six 4 mm plugs in the uppermost expanding middle leaflet of the trifoliate leaf 4 hours after sunrise at the V4 leaf stage (25 days post-seeding), frozen immediately in liquid nitrogen and stored at -80°C until further use (**Figure 1A**). The time points were chosen based on previously published data indicating highest expression of flowering genes four hours after sunrise (Kong et al., 2010; Sun et al., 2011). Furthermore, the V4 stage was determined based on preliminary qRT-PCR analyses of the expression of the flowering genes *Glyma.16G150700* (*FLOWERING LOCUS T 2A*; *GmFT2a*) and *Glyma.16G044100* (*FLOWERING LOCUS T 5A*; *GmFT5a*) in the parental lines (data not shown). To do so, we compared the expression for the V1 to V5 stages and chose the stage which exhibited the highest expression for both of these genes as the *FT* florigens promote the transition to reproductive development and flowering. The extraction and purification of total DNA from leaf tissue was performed using the Omega Bio-Tek Mag-bind Plant Kit and Mag-Bind Total Pure NGS kit (Omega Biotek, Georgia state, USA). Construction of the whole genome sequencing (WGS) libraries for the QS15524_F2:F3_ parental lines was performed by pooling the leaf tissue from the five pots of each parent. Extraction of total DNA and library preparation was performed at the Génome Québec Innovation Centre (Montréal, Canada) using the NxSeq^®^ AmpFREE Library Preparation kit (Lucigen, Wisconsin, U.S.A.). The two parental libraries were combined and sequenced at a 15X depth on the Illumina HiSeq X platform with 150 bp paired-end reads.

The genotyping-by-sequencing (GBS) libraries of the QS15524_F2:F3_ and QS15544_RIL_ mapping populations were prepared using the *PstI/MspI* enzymes as described in Abed et al. (2019) at the Institute of Integrative Biology and Systems (Laval University, Québec, Canada). Sequencing of the QS15524_F2:F3_ GBS libraries was performed by randomly combining a total of 91 barcoded samples per library and by sequencing with four Ion PI V3 chips per library (**Figure 1B**). For the QS15544_RIL_ population, the samples were randomly divided into two sets of 91 samples and sequenced with two Ion PI V3 chips per library. Sequencing for all libraries was performed on the Ion Proton Sequencer and HiQ chemistry at the Institute of Integrative Biology and Systems (Laval University, Québec, Canada).

Total RNA was extracted from samples using a standard Trizol RNA extraction procedure with two extra ethanol rinses to improve purity. Messenger RNA (mRNA) was isolated using the NEBNext mRNA stranded library preparation kit (New England Biolabs, Ontario, Canada) at the Génome Québec Innovation Centre (Montréal, Canada). Two libraries containing 96 pooled samples were prepared per population. Each library was then sequenced on two Illumina NovaSeq6000 S2 (QS15524_F2:F3_) or S4 (QS15544_RIL_) lanes at the Génome Québec Innovation Centre (Montréal, Canada), with four sequencing lanes per population and a total of 8000 M and 9600 M paired-end reads per population, respectively (**Figure 1C**).

### Bioinformatics

The bioinformatic analyses were performed as detailed in Gélinas Bélanger et al. (2024a). Briefly, alignment of all the sequences was performed using version 2 of the *Glycine max* reference genome (Gmax_275_v2.0) (https://phytozome-next.jgi.doe.gov/) (Accessed 8 December 2017; https://data.jgi.doe.gov/). Processing of the WGS sequencing datasets of the QS15524_F2:F3_ parental lines was performed using the fast-WGS pipeline with the default settings (Torkamaneh et al., 2017). The processing of GBS datasets was performed using the fast-GBS pipeline (Torkamaneh et al., 2017) (**Figure 1B**). Variant calling was performed with Platypus version 0.8.1 (Rimmer et al., 2014) with the following commands: –minReads=2, –minMapQual=20 and –minBaseQual=20. Subsequently, a filtering step using vcftools version 0.1.16 (Danecek et al., 2011) was performed with the following parameters: (i) remove non-biallelic sites; (ii) remove InDels; (iii) remove scaffolds; and (iv) filter alleles using the –maxmissing 0.2, –maf 0.3 and –mac 4 commands. Self-imputation was then performed on the missing data for the QS15524_F2:F3_ and QS15544_RIL_ populations using Beagle version 4.1.0 (Browning and Browning, 2007) with twelve iterations. Phasing was then performed with Convert2Map (https://bitbucket.org/jerlar73/convert-genotypes-to-mapping-files/src/master/) using the fast-WGS resequencing data for the QS15524_F2:F3_ parental lines and the GmHapMap dataset for the QS15544 parental lines. A last round of filtering was performed in the QS15544_RIL_ dataset by removing all SNPs with > 10% heterozygous calls before binning with QTL IciMapping (Meng et al., 2015). For QS15524_F2:F3_, the binning step was performed with Genotype Corrector.

Processing of the RNA datasets was performed using multiple publicly available software tools with an in-house script (**Figure 1D**). Briefly, adapters were removed using Trimmomatic version 0.33 (Bolger et al., 2014) with the following options: ILLUMINACLIPTruSeq3-SE.fa:2:30:15, LEADING:3 and TRAILING:3, SLIDINGWINDOW:3:20, and MINLEN:32. Filtered reads were then aligned to the soybean reference transcriptome using TopHat2 version 2.1.1 (Kim et al., 2013). Aligned reads were then counted using HTSeq-count version 0.6.1 (Anders et al., 2015) and were filtered to be considered expressed only if they met the following criteria: (i) min raw counts of at least two to be considered active in a given line; and (ii) transcription recorded in a minimum of 25% of the population. This filtering step resulted in gene sets comprising 38,692 and 40,218 genes for the QS15524_F2:F3_ and QS15544_RIL_ populations, respectively.

### Linkage map construction

The linkage maps were built as described in Gélinas Bélanger et al. (2024a) (**Figure 1B**). Briefly, the maps were generated using QTL IciMapping version 4.2 (Meng et al., 2015) with the Kosambi mapping function. For both maps, the markers were anchored to their physical positions when ordering and the resulting linkage groups (LGs) were split when gaps exceeded 30 cM. The robustness of both linkage maps was previously demonstrated in two previous studies that aimed at mapping reproductive (Gélinas Bélanger et al., 2024a) and seed quality (Gélinas Bélanger et al., 2024b) traits by plotting the (i) genetic distance versus the physical position and (ii) the pairwise recombination fraction and LOD score (**Figure 1B**). In addition, the high-quality of the linkage maps was assessed by confirming the synteny between the physical and genetic positions of the markers (data not shown).

### Measurement of differential gene expression

Measurement of differential gene expression was performed in the QS15524_F2:F3_ (OV vs MA; **Supplementary Table S1A**) and QS15544_RIL_ (MD vs ‘9004’; **Supplementary Table S1B**) parental lines (**Figure 1E**). Each analysis was performed using the early-maturing parent (QS15524_F2:F3,_ OV; QS15544_RIL_, MD) as the reference line. Due to low-quality data in the RNA-seq datasets in the QS15524_F2:F3_ parental lines, two replicates were removed each from the OV and MA samples, thus resulting in a total of three replicates per parent. Similarly, one replicate was removed from the MD and ‘9004’ samples in the QS15544_RIL_ parental lines, resulting in a total of four replicates per parent. The expressed gene sets comprised 38,692 genes for the QS15524_F2:F3_ parents and 40,218 genes for the QS15544_RIL_ parents which were filtered using the aforementioned parameters. Differentially expressed gene analysis was performed in iDEP.96 (Ge et al., 2018) using the DESeq2 function with a false discovery rate (FDR) adjusted p-value threshold fixed at 0.05 and a minimum fold change of 2.0. The normalization of the transcripts for the GO analysis and the eQTL mapping (see below) was performed using the DESeq2 R package (Love et al., 2014). Volcano plots and heatmaps were respectively generated using the online version of VolcaNoseR (Goedhart and Luijsterburg, 2020) and iDEP.96.

### Gene ontology enrichment

Gene ontology (GO) enrichment was performed on the parental downregulated and upregulated gene sets using the Soybase_GOtool (https://www.soybase.org/goslimgraphic_v2/dashboard.php) as detailed in Morales et al. (2013) (**Figure 1E**). The Fisher test p-values obtained with the Soybase_GOtool were adjusted using the Bonferroni correction with a threshold for these corrected p-values fixed at 0.01. In Soybase, the obtained p-value is automatically multiplied by the number of scanned genes (e.g. p*-*value 0.003 X 4000 genes = Bonferroni corrected p-value of 12), leading to p-values that can be above 1. From this list of results, the GO terms associated with molecular functions and cellular components were manually removed using https://biodbnet-abcc.ncifcrf.gov/db/db2db.php, and only the GO terms associated with biological processes were retained. Following this step, we manually curated and retained GO terms associated with the following biological functions from Soybase (Grant et al., 2009): (i) flowering; (ii) reproduction; (iii) senescence; (iv) photosynthesis; and (v) development. This list of GO terms included a total of 162 annotations (**Supplementary Table S1C**) and is herein referred to as FRSPD_GO (Flowering/Reproduction/Senescence/Photosynthesis/Development). In this paper, this list was used to annotate the enriched FRSPD terms in the parental differentially expressed gene (DEG) datasets, mapped eQTL interactions, and genes found in co-expression networks (CEN).

### Expression quantitative trait loci analysis

Transcriptome-wide eQTL analysis was performed on normalized transcript abundances for the 176 lines of the QS15524_F2:F3_ population (38,692 genes) and the 162 lines of the QS15544_RIL_ (40,218 genes) population (**Figure 1F**). The mapping of eQTL was performed using a combinatorial approach which includes the use of three different algorithms: (i) Inclusive composite interval mapping (ICIM) approach implemented in QTL IciMapping version 4.2 (Meng et al., 2015); (ii) Interval mapping (IM) from QTL IciMapping version 4.2 (Meng et al., 2015); and (iii) Genome-wide compositive interval mapping (GCIM) method in the QTL.gCIMapping.GUI.v2.0.GUI package (Zhang et al., 2020). The LOD thresholds for ICIM and IM were calculated in QTL IciMapping with 1000 permutations using an α of 0.05 and a walking step of 1 cM for genome-wide scanning. To limit the computational burden (i.e. at least 1,000 permutations for 38,692 and 40,218 genes), we performed permutations on 100 randomly sampled gene transcripts (i.e. 1,000 permutations X transcripts for 100 randomly selected genes = 100,000 permutations) as performed in West et al. (2007); Wang et al. (2010, 2014), and Huang et al. (2020). Subsequently, the global permutation threshold was calculated as the 95^th^ percentile of the representative null distribution and equaled to (i) 4.01 for ICIM in QS15544_RIL_; (ii) 3.99 for IM in QS15544_RIL_; (iii) 4.13 for ICIM in QS15524_F2:F3_; and (iv) 4.12 for IM in QS15524_F2:F3_. For GCIM, the fixed model component was chosen for the QS15544_RIL_ population and the fixed-restricted maximum likelihood (REML) component was chosen for the QS15524_F2:F3_ population, both with a walking speed of 1 cM. In the QTL.gCIMapping.GUI.v2.0.GUI package, the likelihood function is only available for F_2_ populations and was chosen based on prior testing. For GCIM, the default LOD threshold suggested in the literature is 2.5 for QTL studies; however, the thresholds were increased to 7.5 for the QS15524_F2:F3_ and 4.0 for the QS15544_RIL_ populations to reduce the noise and remove minor eQTL interactions. The contrasting LOD thresholds for the QS15524_F2:F3_ and QS15544_RIL_ populations were chosen based on preliminary tests performed using the different functions implemented in the QTL.gCIMapping.GUI.v2.0.GUI package. Following the mapping of interactions with the three algorithms, all of the significant interactions were classified either as *cis*-acting or *trans*-acting. Interactions were classified as *cis*-acting if within 1,000,000 bp region from the transcription start site (TSS) of the studied gene, whereas interactions were considered *trans*-acting if identified outside this 1,000,000 bp region or on another chromosome.

To increase our confidence in the eQTL regions identified by the three methods, only signals identified by at least two methods and within 1 Mbp of each other were retained. To do so, the interactions were split between *cis*-acting and *trans*-acting, and the size of each of the mapped eQTL regions (i.e. all of the interactions identified with the three aforementioned algorithms) was manually adjusted by adding 500,000 bp both upstream and downstream of the loci. The overlapping regions were subsequently identified using the genomic peak Venn function implemented in https://www.bioinformatics.com.cn/en, a free online platform for data analysis and visualization. To compute the interactions using this software, each interaction was codified as the following: cis/trans_genename_interactingchromosome startregioninteraction endregioninteraction. For example, *trans_Glyma.01G123600_GM05 40000 200000* would represent the region 40,000 – 200,000 on chromosome GM05 interacting in *trans* with *Glyma.01G123600*. The overlaps were identified using a pairwise comparison using the ICIM interactions as the reference signals in the ICIM vs. IM and ICIM vs. GCIM analyses. In addition, the IM signals were used as references in the IM vs. GCIM analysis. *Trans* interactions overlapping *cis* regions were *de facto* considered as *cis*.

### Regulatory hotspot mapping

To uncover regions associated with the regulation of the expression of multiple genes, we decided to identify the hotspots involved in the modulation of a high number of *trans* interactions (**Figure 1F**). To do so, marker pairs delineating *trans* hotspots were qualified based on their respective (i) number of *trans* interactions and (ii) *trans* interaction density, and only those meeting both of these criteria were considered as markers flanking a hotspot region. The number of interactions was identified by summing the number of *trans* interactions associated with a specific pair of markers and only the pairs of markers that were above the 95^th^ (minor hotspot) or the 99^th^ (major hotspot) percentiles threshold of all marker pairs from that population were retained. The *trans* interaction density was quantified by identifying the average number of *trans* interactions per kbp associated with the distance between the flanking of markers. A specific marker pair was deemed significant if its density was above the 80^th^ percentile of all of the calculated *trans*-interaction densities. To facilitate the reading and understanding of the paper, each of the loci presented in this article is distinguished using either F2 (QS15524_F2:F3_) or RIL (QS15544_RIL_) in front of the region’s name (e.g. F2_GM18:1,911,667-1,935,386). All of the Circos plots present in this paper were drawn using Circa V1 https://omgenomics.gumroad.com/l/circa.

### Co-expression network analysis and identification of homologous genes using protein homology

Co-expression networks were built for the target genes and candidate TFs to understand their global expression pattern within the transcriptome (**Figure 1G**). To understand the general expression pattern of the interactions associated with a specific hotspot, we generated the pairwise Pearson correlation coefficients (PCC) for the queried genes and clustered them using the pheatmap package (https://github.com/raivokolde/pheatmap) implemented in R. Transcriptome-wide CENs (TWCENs) were also generated using the QS15524_F2:F3_ (38,692 genes) and QS15544_RIL_ (40,218 genes) expression datasets. To do so, PCCs were generated using ≥ 0.85 (positive TWCEN, herein named POS_TWCEN_) or ≤ -0.85 (negative TWCEN, herein named NEG_TWCEN_) as thresholds for the expression datasets for the 176 and 162 lines for QS15524_F2:F3_ and QS15544_RIL_, respectively. The genes deemed significant based on these thresholds were then annotated using the Soybase_GOtool to identify FRSPD_GO functions. Identification of homologous genes was performed using their peptide sequence with the Blast function in Phytozome V13 (Goodstein et al., 2012), and peptide sequences exhibiting an E ≤ 1e-5 were considered homologous.

### Prediction of transcription factors and identification of candidate single nucleotide polymorphisms

Following the mapping of eQTL interactions and regulatory hotspots, we predicted putative transcription factors that could be regulators for the four candidate genes (*GmPRR1a*, *GmMDE04*, *GmLHCA4a*, and *GmCDF3*) of the *E8-r3* locus, a region previously identified between the GM04:41,808,599 and GM04:42,376,23 flanking markers (**Figure 1H**; **Supplementary Table S1D**) (Gélinas Bélanger et al., 2024a). To do so, we generated a list of 4,611 putative TFs (herein named TF_list_4,611_) (**Supplementary Table S1E**). The TF_list_4,611_ was generated by merging the genes with annotated TF functions from the PlantTFDB (Jin et al., 2017) and Soybase (Grant et al., 2009) databases. This corresponded to a total of 658 and 864 unique genes from the PlantTFDB and Soybase, respectively. In addition, another common 3,089 genes were identified in both databases. To identify the best candidate TFs present in the identified loci, we subsequently annotated each of them using the (i) differential transcript expression datasets from the parents; (ii) positive and negative co-expression network datasets; and (iii) Soybase gene ontology annotations.

In addition, we used a custom variant analysis pipeline similar to the one detailed in Gélinas Bélanger et al. (2024a) to identify putative causal mutations in the predicted TFs (**Figure 1I**). Prediction of deleterious effects of the SNPs within these TFs was performed using Ensembl Variant Effect Predictor (VEP) with Glycine_max_v2.1 (McLaren et al., 2016). Mutations predicted as having moderate or high consequences on the protein structure or located in the 3’UTR/5’UTR were retained, whereas the others were removed from the dataset. The putative effects of the identified missense mutations were then predicted using Sorting Intolerant From Tolerant 4G (SIFT4g) (Ng and Henikoff, 2003; Kumar et al., 2009). To predict the effects of these mutations, we generated a database using the annotations of *G. max* Wm82.a2.v1 from EnsemblPlants and the SIFT4G_Create_Genomic_DB guidelines https://github.com/pauline-ng/SIFT4G_Create_Genomic_DB. Single nucleotide polymorphisms with SIFT scores ≥ 0.05 were considered as tolerated and those < 0.05 were considered as deleterious. Following the identification of the candidate SNPs, we verified the genotypes associated with them using the GmHapMap dataset and retained only the SNPs that were present in a single parental line.

## Results

### Linkage map construction and differential gene expression the parental lines

In our previous QTL study (Gélinas Bélanger et al., 2024a), 541,106,451 (QS15524_F2:F3_) and 286,844,986 (QS15544_RIL_) unique single-end reads were generated during the sequencing step of the full mapping populations. After filtering, two linkage maps were generated from 1,613 (QS15524_F2:F3_; **Supplementary Tables S1F, G**) and 2,746 (QS15544_RIL_; **Supplementary Tables S1F, H**) high-quality GBS-derived SNP markers. To validate our choice of experimental conditions for both of our populations (i.e. RNA collected from the middle leaflet of the trifoliate leaf 4 hours after sunrise at the V4 leaf stage), we performed a differential gene expression analysis in both pairs of parental lines. Based on this analysis, we identified 10,216 DEGs (4,953 up-regulated genes and 5,263 down-regulated genes in OV) in the QS15524_F2:F3_ parents (**Supplementary Figures S1A, B**; **Supplementary Table S1I**) and 1,430 DEGs (438 upregulated genes and 992 down-regulated genes in MD) in the QS15544_RIL_ parents (**Supplementary Figures S1C, D**; **Supplementary Table S1J**). To find an explanation to the large difference of DEGs between each population, we inspected the pedigrees of QS15524_F2:F3_ and QS15544_RIL_ but did not find any obvious factors (e.g., a cross using a very exotic line) that would have caused this discrepancy (data not shown). Overall, we found that two of our candidate genes, *GmCDF3* and *GmMDE04*, were upregulated in the OV parental line of the QS15524_F2:F3_ population. In addition, we found that many FRSPD_GO terms were significantly enriched for both populations (e.g. ‘Regulation of Flower Development’ in the QS15524_F2:F3_ parents, Bonferroni corrected p-value of 3.05E-76), thus indicating a large difference in the abundance of transcripts of FRSPD genes in the parental lines of both populations (**Supplementary Table S1K**).

### Mapping of eQTL interactions

**Figure 2:**
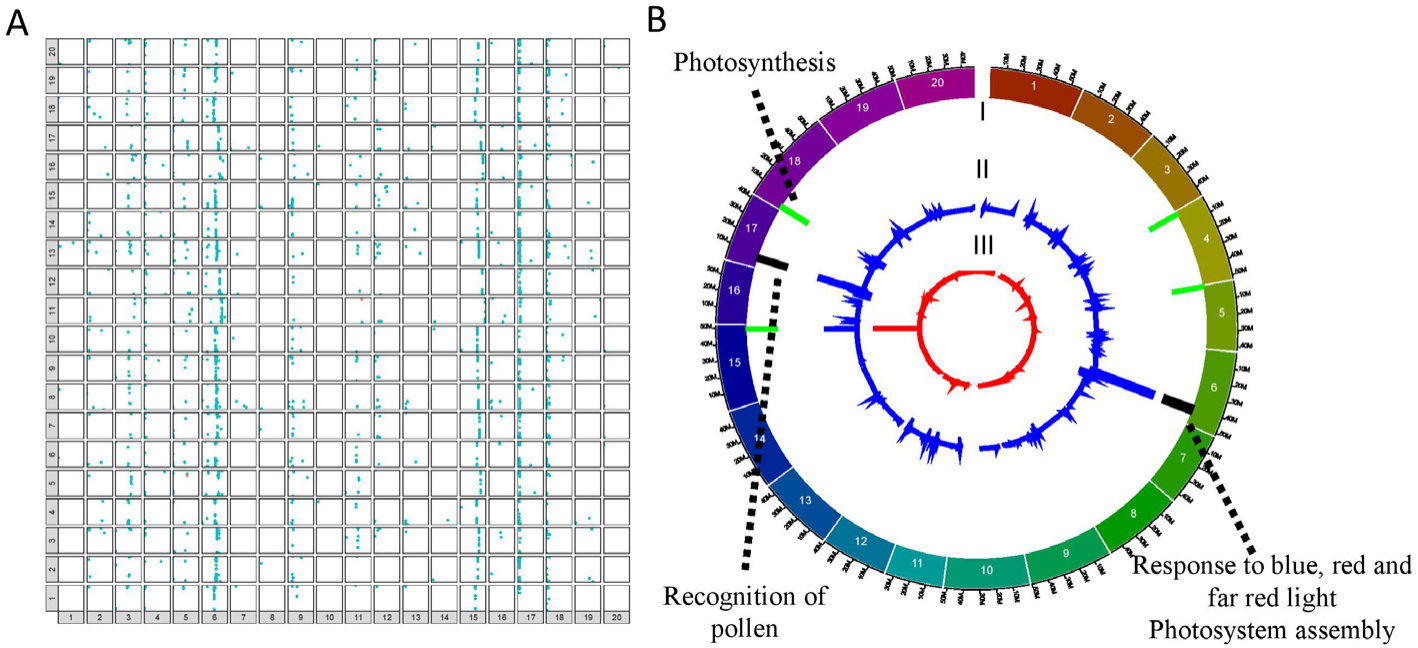
Mapping of the eQTL interactions and regulatory major hotspots using the combinatorial approach in the QS15524_F2:F3_ population. **(A)** Identification of the eQTL interactions found with at least two mapping algorithms in the QS15524_F2:F3_ population. The X-axis represents regulating regions, whereas the Y-axis represents the locations of the target genes. *Cis* interactions and *trans* interactions are respectively illustrated as the orange and light blue dots. **(B)** Mapping of the regulatory hotspots. Level I, locations of the hotspots. Major and minor hotspots are respectively indicated using black and green rectangles. Level II, number of eQTL interactions per marker. Level III, eQTL density per marker. The dotted lines indicate the FRSPD_GO functions significantly associated with the hotspots (Bonferroni p-value, 0.01).

**Figure 3:**
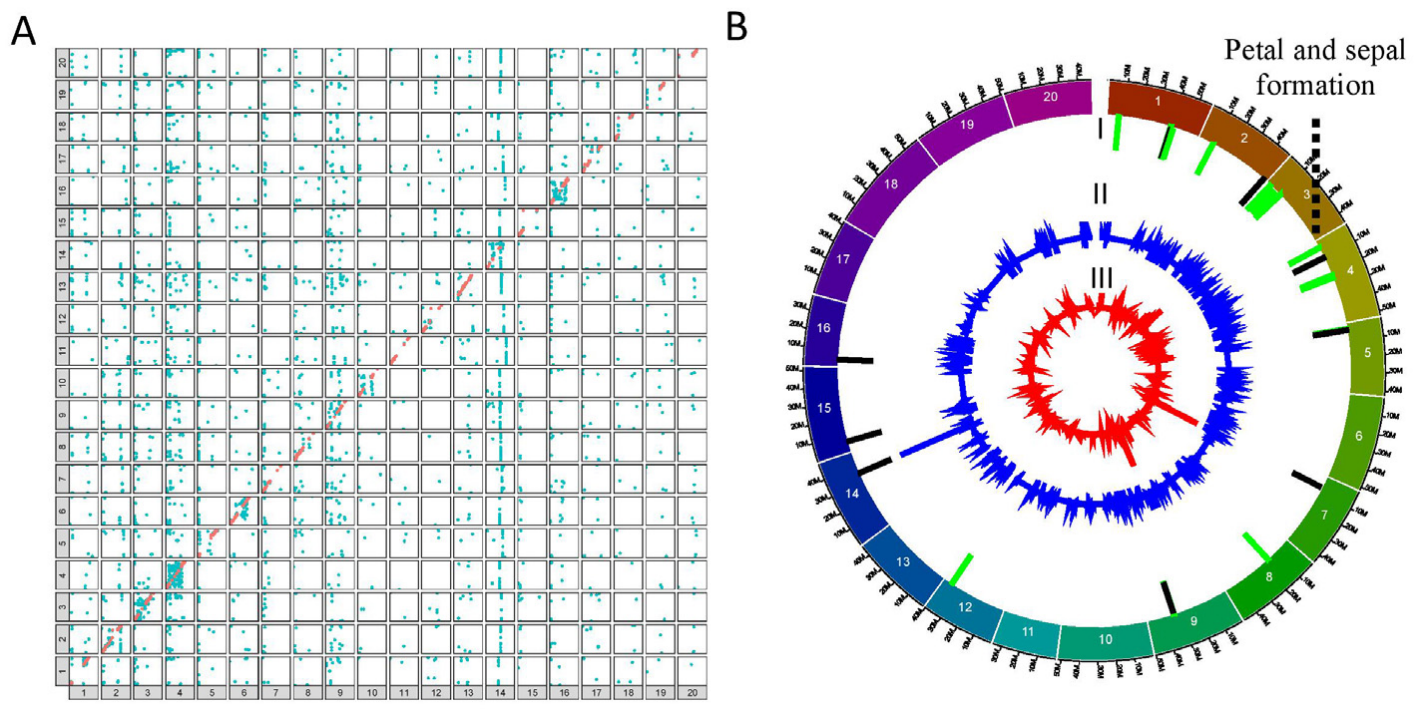
Mapping of the eQTL interactions and regulatory major hotspots using the combinatorial approach in the QS15544_RIL_ population. **(A)** Identification of the eQTL interactions found with at least two mapping algorithms in the QS15544_RIL_ population. The X-axis represents regulating regions, whereas the Y-axis represents the locations of the target genes. *Cis* interactions and *trans* interactions are respectively illustrated as the orange and light blue dots. **(B)** Mapping of the regulatory hotspots. Level I, locations of the hotspots. Major and minor hotspots are respectively indicated using black and green rectangles. Level II, number of eQTL interactions per marker. Level III, eQTL density per marker. The dotted lines indicate the FRSPD_GO functions significantly associated with the hotspots (Bonferroni p-value, 0.01).

Subsequently, the linkage maps were used to perform genome-wide mapping of eQTL interactions for the QS15524_F2:F3_ (38,693 genes) and QS15544_RIL_ (40,223 genes) populations using a combinatorial approach based on the IM, ICIM, and GCIM algorithms populations using acombinatorial approach based on the IM, ICIM, and GCIMalgorithms (**Supplementary Table S1L**; **S1m**). In the QS15524_F2:F3_ population, the ICIM (4,735 *trans*/17 *cis*), IM (1,714 *trans*/10 *cis*), and GCIM (10,906 *trans*/32 *cis*) methods identified a varying number of interactions (**Table 1**). The same analysis was performed with the QS15544_RIL_ population, with IM (17,375 *trans*/5,337 *cis*) having the highest number of interactions followed by ICIM (7,941 *trans*/2,862 *cis*) and then GCIM (4,418 *trans*/2,375 *cis*) (**Table 1**). To reduce the number of regions for further analyses, we decided to retain only interactions that were identified by at least two algorithms and which were overlapping or within a 1,000,000 bp distance from each other (**Table 1**; **Supplementary Table S1N**, **S1o**, **S1p**). This merging step reduced the number of interactions to 2,218 *trans* (2,061 genes)/7 *cis* (7 genes) (**Figure 2A**) and 4,073 *trans* (2,842 genes)/3,083 *cis* (2,418 genes) (**Figure 3A**) for the QS15524_F2:F3_ and QS15544_RIL_ populations, respectively (**Supplementary Table S1Q**). Using our combinatorial approach, we identified that the *trans* interactions were regulated by a total of 280 regions covering a total of ≈ 212.19 Mbp in the QS15524_F2:F3_ population and 1,213 regions covering a total of ≈ 588.03 Mbp in the QS15544_RIL_ population. The number of interactions per region was between 1 and 507 with a density between 3.67E-5 and 1,481 interactions/kbp for the QS15524_F2:F3_ population. For the QS15544_RIL_ population, the number of interactions per region was between 1 and 450 with a density between 3.09E-7 and 100 interactions/kbp.

**Table 1:** Number of eQTL interactions and eQTL regions before and after the merge using the genomic peak Venn function.

Population	Method	Number of eQTL interactions		Merging of the eQTL regions using the genomic peak Venn function				
				Methods involved in the merging	Number of regions with duplicates^1^	Number of unique regions (without duplicates)^2^	Number of regions with duplicates^1^	Number of unique regions (without duplicates)^2^
		*Trans*	*Cis*		*Trans*		*Cis*	
**QS15524_F2:F3_**	**ICIM**	4,735	17	**ICIM vs IM**	1,340		7	
	**IM**	1,714	10	**ICIM vs GCIM**	1,134		1	
	**GCIM**	10,906	32	**IM vs GCIM**	31		1	
	**Total**	**17,355**	**59**	**Total**	2,505	**2,218**	9	**7**
**QS15544_RIL_**	**ICIM**	7,941	2,862	**ICIM vs IM**	2,058		1,046	
	**IM**	17,375	5,337	**ICIM vs GCIM**	1,796		2,302	
	**GCIM**	4,418	2,375	**IM vs GCIM**	1,649		1,046	
	**Total**	**29,734**	**10,574**	**Total**	5,503	**4,073**	4,394	**3,083**

^1^ Number of eQTL regions after the merge with the genomic peak Venn function. The total number includes all of the duplicated regions between the methods.

^2^ Number of eQTL regions after the merge with the genomic peak Venn function but without the duplicates found in each of the merging steps.

### Identification and characterization of the hotspots and regions associated with *E8-r3*

To identify important regulatory regions controlled by or controlling the *E8-r3* region, we began by classifying our *trans* eQTL regions into minor regions or hotspots (either minor or major hotspots) to identify the most promising regions (**Supplementary Table S1R**). To do so, we retrieved all of the regions above the 95^th^ (minor hotspot) or 99^th^ (major hotspot) percentiles for the number of interactions and the 80^th^ percentile for the eQTL interaction density to identify either minor or major hotspots (**Table 2**). For the QS15524_F2:F3_ population, the thresholds corresponded to 29 to 259 interactions for minor hotspots or ≥ 260 interactions for major hotspots with densities ≥ 0.09 interactions/kbp. For the QS15544_RIL_ population, the thresholds corresponded to 9 to 16 interactions for minor hotspots or ≥ 17 interactions for major hotspots with densities ≥ 0.17 interactions/kbp. Using these thresholds, 8 hotspots (2 major and 6 minor) were identified in the QS15524_F2:F3_ population (i.e. 2.85% of the total number of identified regions) (**Figure 2B**; **Table 2**). Similarly, 34 hotspots (9 major and 25 minor) were identified in the QS15544_RIL_ population (i.e. 1.23% of the total number of identified regions), with a large number of hotspots located near each other on chromosomes GM01, GM03, GM04, GM05 and GM09 (**Figure 3B**; **Table 2**). Following the identification of the hotspots, we noticed that several had markers in common, suggesting that these regions might be regulated by one or several common loci. This included the (i) F2_GM18:1,434,182-1,911,667 and F2_GM18:1,911,667-1,935,386 and the (ii) F2_GM15:49,385,092-49,442,075 and F2_GM15:49,442,075-49,442,237 regions of the QS15524_F2:F3_ population. Due to their close location, we merged the neighboring loci into two merged regions named F2_GM18:1,434,182-1,935,386 and F2_GM15:49,385,092-49,442,237 for the subsequent analyses. To characterize the hotspots, we subsequently performed a GO enrichment analysis on each of them and observed that four (i.e. three in QS15524_F2:F3_ and one in QS15544_RIL_) were significantly enriched with terms associated with FRSPD functions (**Figures 2B**, **3B**; **Supplementary Table S1S**).

**Table 2:** Major and minor hotspots in the QS15524_F2:F3_ and QS15544_RIL_ populations.

Population	Chromosome	Start of region (bp)	End of region (bp)	Size of region (bp)	Nb of interactions	Density (interactions/kbp)	Percentile
QS15524_F2:F3_	GM06	39,892,719	43,437,125	3,544,406	507	0.14	Major (99^th^)
	GM17	5,431,473	7,260,313	1,828,840	342	0.19	
QS15544_RIL_	GM01	39,404,966	39,405,971	1,005	17	16.92	Major (99^th^)
	GM02	46,648,011	46,714,310	66,299	80	1.21	
	GM04	10,812,813	10,985,437	172,624	77	0.45	
	GM05	3,769,727	3,884,649	114,922	38	0.33	
	GM07	6,889,969	6,890,075	106	35	330.19	
	GM09	34,116,171	34,117,683	1,512	18	11.91	
	GM14	35,854,652	35,899,383	44,731	450	10.06	
	GM15	6,116,797	6,146,533	29,736	20	0.67	
	GM16	3,627,910	3,667,696	39,786	17	0.43	
QS15524_F2:F3_	GM04	1,404,047	1,408,537	4,490	48	10.70	Minor (95^th^)
	GM05	208,889	294,855	85,966	46	0.54	
	GM15	49,385,092	49,442,075	56,983	53	0.93	
	GM15^1^	49,442,075	49,442,237	162	240	1,480.00	
	GM18	1,434,182	1,911,667	477,485	51	0.11	
	GM18^2^	1,911,667	1,935,386	23,719	40	1.69	
QS15544_RIL_	GM01	6,517,814	6,579,997	62,183	11	0.18	Minor (95^th^)
	GM01	6,580,209	6,580,306	97	10	103.09	
	GM01	39,112,506	39,154,217	41,711	10	0.24	
	GM01	39,399,889	39,404,966	5,077	10	1.97	
	GM01	40,907,974	40,908,162	188	12	63.83	
	GM02	8,822,628	8,834,850	12,222	9	0.74	
	GM03	2,411,109	2,411,229	120	10	83.33	
	GM03	2,411,229	2,450,947	39,718	16	0.40	
	GM03	3,038,816	3,039,628	812	12	14.78	
	GM03	6,809,980	6,810,064	84	9	107.14	
	GM03	10,811,705	10,834,554	22,849	14	0.61	
	GM03	10,834,651	10,836,440	1,789	15	8.39	
	GM04	4,093,911	4,101,799	7,888	10	1.27	
	GM04	4,158,205	4,185,355	27,150	16	0.59	
	GM04	22,705,951	22,709,551	3,600	15	4.17	
	GM04	23,570,549	23,642,207	71,658	14	0.20	
	GM04	24,217,772	24,253,427	35,655	9	0.25	
	GM05	3,081,540	3,124,214	42,674	11	0.26	
	GM05	3,167,692	3,167,836	144	15	104.17	
	GM05	3,583,876	3,584,733	857	12	14.00	
	GM08	16,115,503	16,148,760	33,257	10	0.30	
	GM09	33,937,720	33,971,428	33,708	9	0.27	
	GM09	33,971,428	33,990,878	19,450	16	0.82	
	GM09	34,060,804	34,116,171	55,367	9	0.16	
	GM12	35,211,026	35,246,755	35,729	16	0.45	

^1^ Interacts with *GmMDE04* and *GmPRR1a*.

^2^ Interacts with *GmLHCA4a*.

As we were interested in understanding the role of the *E8-r3* region and its interactions, we investigated whether the identified eQTL minor regions and eQTL hotspots interacted in *trans* with our four candidate genes (*GmCDF3*, *GmPPR1a*, *GmLHCA4a*, and *GmMDE04*) (**Table 3**). On the whole, we identified that the F2_GM18:1,434,182-1,935,386 hotspot was involved in the regulation of *GmLHCA4a*. We also detected that *GmPPR1a* was regulated by the F2_GM15:49,385,092-49,442,237 hotspot as well as the RIL_GM04:17,227,512-20,251,662, RIL_GM04:31,408,946-31,525,671 and RIL_GM13:37,289,785-38,620,690 minor regions. For *GmMDE04*, we identified one interaction with the F2_GM15:49,385,092-49,442,237 hotspot and one interaction with the RIL_GM04:17,227,512-20,251,662 minor region. No interactions were observed for *GmCDF3*, and as such, this gene was not investigated further. In addition to the interactions with the *E8-r3* candidate genes, we also identified several *trans* regulatory events with five additional genes (*Glyma.04G168100*, *Glyma.04G168000*, *Glyma.04G169300*, *Glyma.04G168200*, and *Glyma.04G169100*) found in the *E8-r3* locus, including several loci found on GM04 (**Supplementary Table S1Q**).

**Table 3:** Expression quantitative trait loci for the *GmLHCA4a*, *GmPRR1a*, and *GmMDE04* genes.

Region	Method	Gene Name	Linkage group	Marker		LOD	PVE (%)	Additive effect	Dominance effect
				Left marker	Right marker				
F2_GM15:49,442,075-49,442,237^1^	ICIM	*GmPRR1a*	15	49,442,075	49,442,237	4.3	10.7	2.1	-57.0
	IM	*GmPRR1a*	15	49,442,075	49,442,237	4.3	10.7	2.1	-57.0
	GCIM	*GmMDE04*	15	49,442,075	49,442,237	17.1	15.0	0	101.2
	ICIM	*GmMDE04*	15	49,442,075	49,442,237	4.2	8.7	1.1	96.6
F2_GM18:1,911,667-1,935,386^2^	GCIM	*GmLHCA4a*	18	1,911,667	1,911,667	9.8	5.3	0	6,454.1
	ICIM	*GmLHCA4a*	18	1,911,667	1,935,386	4.5	8.0	1,204.7	8,147.8
RIL_GM04:17,227,512-20,251,662^3^	ICIM	*GmMDE04*	4	17,227,512	17,230,775	6.6	14.7	-97.6	N/A
	IM	*GmMDE04*	4	17,227,512	17,230,775	5.3	0.8	-89.9	N/A
	GCIM	*GmPRR1a*	4	17,534,130	17,914,073	9.7	19.1	-42.6	N/A
	IM	*GmPRR1a*	4	18,383,138	20,251,662	6.3	1.4	38.8	N/A
RIL_GM04:31,408,946-31,525,671	ICIM	*GmPRR1a*	4	31,408,946	31,525,671	10.1	19.9	42.6	N/A
	IM	*GmPRR1a*	4	31,408,946	31,525,671	6.6	1.5	39.4	N/A
RIL_GM13:37,289,785-38,620,690^4^	GCIM	*GmPRR1a*	13b	37,289,785	37,516,022	6.3	13.6	36.0	N/A
	ICIM	*GmPRR1a*	13b	37,790,482	37,795,923	5.2	9.5	-31.0	N/A
	IM	*GmPRR1a*	13b	38,027,686	38,620,690	5.8	1.4	-40.0	N/A

^1^ The region identified here is F2_GM15:49,442,075-49,442,237 but has been merged to the F2_GM15:49,385,092-49,442,075 region to generate the F2_GM15:49,385,092-49,442,237 hotspot.

^2^ The region identified here is the F2_GM18:1,911,667-1,935,386 but has been merged to the F2_GM18:1,434,182-1,911,667 region to generate the F2_GM18:1,434,182-1,935,386 hotspot.

^3^ The RIL_GM04:17,227,512-20,251,662 region was obtained by merging the farthest left (GM04: 17,227,512) and right (GM04:20,251,662) markers.

^4^ The RIL_GM13:37,289,785-38,620,690 region was obtained by merging the farthest left (GM04: 37,289,785) and right (GM04:38,620,690) markers.

N/A, not available.

### The F2_GM18:1,434,182-1,935,386 hotspot regulates *GmLHCA4a* and several homologous genes

**Figure 4:**
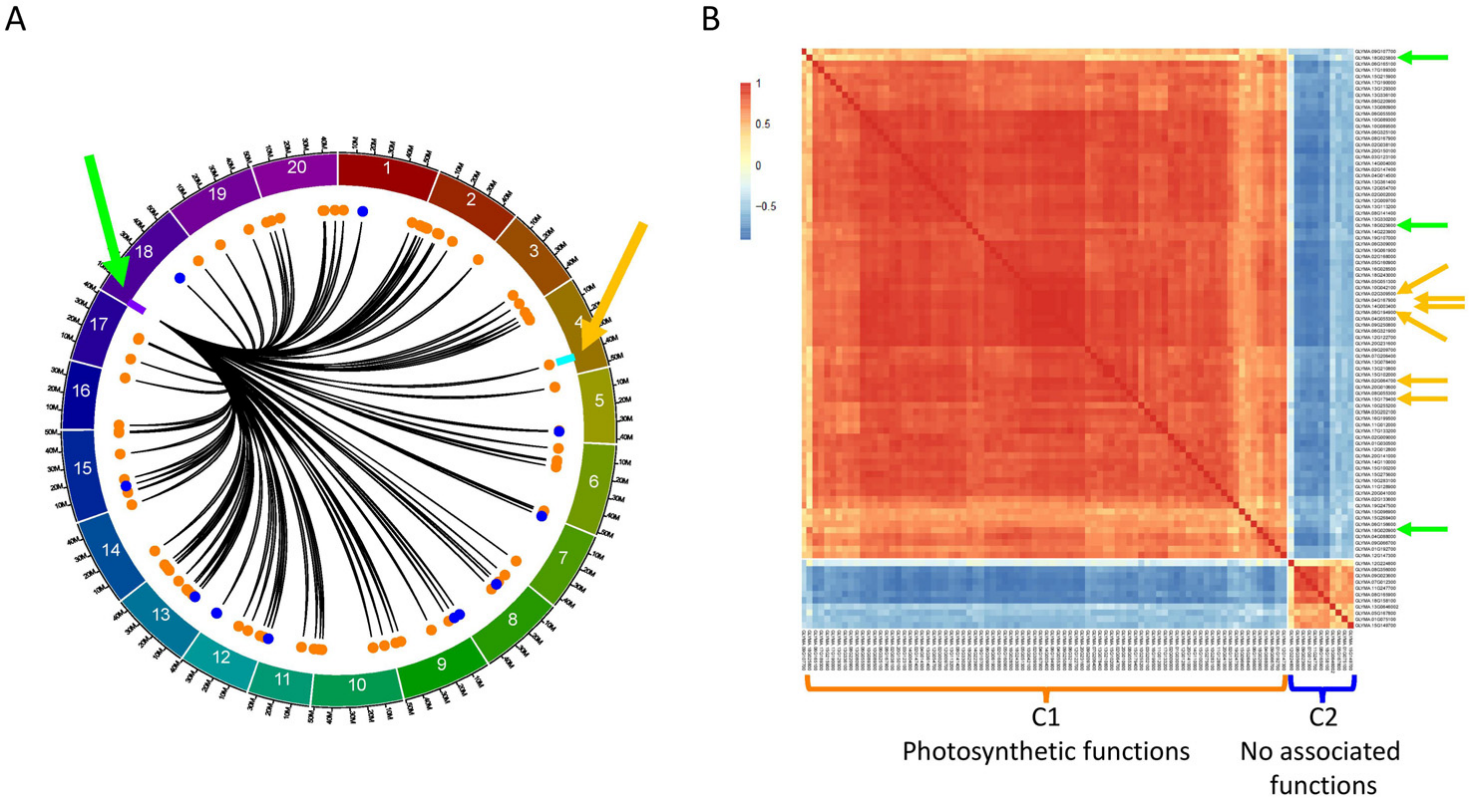
Characterization of the F2_GM18:1,434,182-1,935,386 hotspot and its interaction with *GmLHCA4a* in the QS15524_F2:F3_ population. **(A)** Identification of the 91 *trans* interactions (90 genes) associated with the F2_GM18:1,434,182-1,935,386 hotspot. Colored dots represent the C1 (orange color; 79 genes) and C2 (royal blue color; 11 genes) clusters depicted in panel **(B)** Orange arrow, location of the candidate gene *GmLHCA4a* and the *E8-r3* locus (light blue rectangle). Green arrow, location of the three candidate TFs (*Glyma.18G020900*, *Glyma.18G025600*, and *Glyma.18G025800*) and the F2_GM18:1,434,182-1,935,386 hotspot (purple rectangle). **(B)** Co-expression network with the 90 target genes and three candidate TFs. Orange arrows, location of the six *LHCA* genes (*GmLHCA4a*, *Glyma.02G064700*/*GmLHCA1*, *Glyma.02G309500*/*GmLHCA3a*, *Glyma.06G194900*/*GmLHCA4b*, *Glyma.14G003400*/*GmLHCA3b*, and *Glyma.15G179400*/*GmLHCA6*). Green arrows, location of the three candidate TFs. The C1 cluster is significantly associated with photosynthetic functions, whereas C2 is not enriched with any FRSPD_GO terms.

To further understand the role of the F2_GM18:1,434,182-1,935,386 hotspot, we investigated to understand the specific FRSPD_GO functions of the 91 interactions (90 genes^1^). In addition, we used the TF_list_4,611_ to identify candidate transcription factors and found three (*Glyma.18G020900*, *Glyma.18G025600*, and *Glyma.18G025800*) that were located within or close to this hotspot (**Figure 4A**). To understand the co-expression patterns between the 90 target genes and three candidate transcription factors, we generated a CEN using pairwise PCCs between these 93 genes (**Figure 4B**).

By doing so, we observed that 79 of the target genes, including *GmLHCA4a*, exhibited a similar co-expression pattern and were grouped as such into the F2_GM18:1,434,182-1,935,386_C1 cluster, a group specifically enriched with terms related to photosynthesis and response to light stimulus. Another group comprising 11 target genes was grouped in the F2_GM18:1,434,182-1,935,386_C2 cluster, a group without significantly enriched functions. We found that the three candidate TFs that were identified for the F2_GM18:1,434,182-1,935,386 hotspot all clustered in the C1 group, with *Glyma.18G025600* exhibiting the highest co-expression values with *GmLHCA4a*. Interestingly, we also discovered that the C1 cluster comprised a total of six *LHCA* homologs annotated with photosynthesis, response to light, photosystem I, and chlorophyll-binding functions in Soybase: (i) *Glyma.04G167900*/*GmLHCA4a* (our candidate gene); (ii) *Glyma.02G064700*/*GmLHCA1*; (iii) *Glyma.02G309500*/*GmLHCA3a*; (iv) *Glyma.06G194900*/*GmLHCA4b*; (v) *Glyma.14G003400*/*GmLHCA3b*; and (vi) *Glyma.15G179400*/*GmLHCA6*.

### Functional investigation and variant analysis of the candidate transcription factors regulating *LHCA* homologs

**Figure 5:**
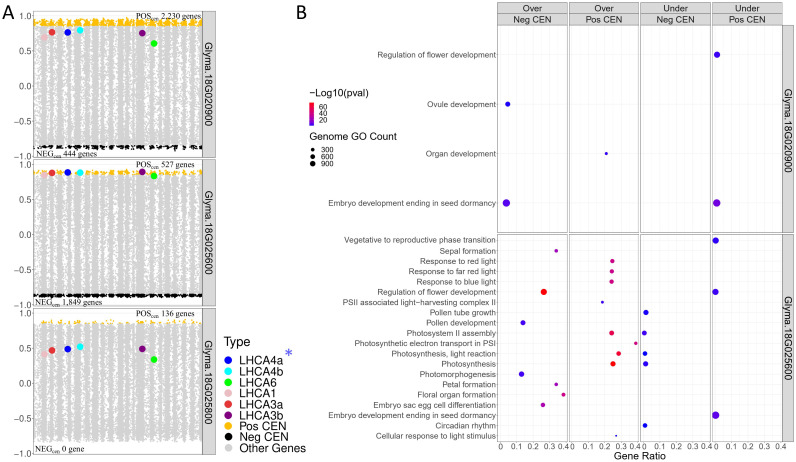
Transcriptome-wide co-expression network for the three candidate TFs of the F2_GM18_1,434,182-1,935,386 hotspot. **(A)** Positive and negative TWCENs for the three candidate TFs using PCC thresholds of ≥ 0.85 (POS_TWCEN_) and ≤ -0.85 (NEG_TWCEN_). As shown in the panel, *Glyma.18G020900* exhibits the largest POS_TWCEN_ (2,230 genes) followed by *Glyma.18G025600* (527 genes), and *Glyma.18G025800* (136 genes). For the NEG_TWCEN_, *Glyma.18G025600* (1,849 genes) displays the largest network, whereas the network of *Glyma.18G020900* is smaller (444 genes). No gene was found for the NEG_TWCEN_ of *Glyma.18G025800*. The highest level of co-expression between the *LHCA* homologs and a candidate TF was achieved with *Glyma.18G025600* with a mean PCC of 0.87 for the six homologs. In comparison, the mean PCC of *Glyma.18G020900* and *Glyma.18G025800* for these six homologs were 0.73 and 0.45, respectively. *GmLHCA4a*, the candidate target gene for the *E8-r3* locus, is highlighted with an asterisk. **(B)** Functional annotation of the POS_TWCEN_ and NEG_TWCEN_ of each candidate TF. The POS_TWCEN_ and NEG_TWCEN_ of the *Glyma.18G025600* gene were significantly enriched with a large number of FRSPD genes associated with photosynthetic properties such as light response. Only gene annotations that are either over-represented (i.e., “Over” facet) or under-represented (i.e., “Under” facet) are displayed in the figure. Non-FRSPD annotations were not displayed for visualization purposes, but are available in **Supplementary Table S1U**.

After identifying the three candidate TFs, we found that these genes were not annotated with FRSPD functions in Soybase. To gain further insights about them, we investigated the TWCENs between these genes and the 38,692 genes dataset from the QS15524_F2:F3_ population (**Figure 5A**; **Supplementary Table S1T**). Using a PCC threshold of ≥ 0.85 (POS_TWCEN_), we found that the POS_TWCEN_ of *Glyma.18G020900*, *Glyma.18G025600* and *Glyma.18G025800* respectively comprised 2,230, 527 and 136 genes. For these three candidate TFs, we also constructed the NEG_TWCEN_ using a PCC threshold of ≤ -0.85 and discovered that the NEG_TWCEN_ of *Glyma.18G020900*, *Glyma.18G025600* and *Glyma.18G025800* respectively comprised 444, 1,849 and 0 genes. The genes found in POS_TWCEN_ and NEG_TWCEN_ of *Glyma.18G025600* were significantly enriched with functions associated with flower development, photosynthesis, and chlorophyll-binding, whereas the POS_TWCEN_ and NEG_TWCEN_ of *Glyma.18G020900* were less strongly associated with these functions (**Figure 5B**; **Supplementary Table S1U**). For *Glyma.18G025800*, we found that its POS_TWCEN_ and NEG_TWCEN_ were not significantly enriched with any GO terms.

To further understand the putative roles of the three candidate TFs located in the F2_GM18:1,434,182-1,935,386_C1 cluster, we investigated their expression profiles, as well as the presence of mutations, in the parental lines. Based on our observations, *Glyma.18G020900* (Fold change, 3.66; FDR adjusted p-value, 1.93E-08) and *Glyma.18G025800* (Fold change, 2.17; FDR adjusted p-value, 0.047), were found to be significantly upregulated in ‘OAC Vision’, whereas *Glyma.18G025600* was not differentially expressed (Fold change, 1.45; FDR adjusted p-value, 0.07) (**Supplementary Figure S2**; **Supplementary Table S1I**). Deeper investigations using our candidate SNP identification pipeline led to the identification of two SNPs in *Glyma.18G025600*, but none of the other candidate TFs (**Table 4**). Overall, the presence of variants in the 3’UTR of *Glyma.18G025600* but not in the other candidates, the high co-expression values between this candidate and all of the *LHCA* homologs, and the FRSPD functions associated with its POS_TWCEN_ and NEG_TWCEN_ suggest that *Glyma.18G025600* is the most likely candidate for the F2_GM18:1,434,182-1,935,386 hotspot.

**Table 4:** Single nucleotide polymorphisms for the candidate transcription factors of the QS15524_F2:F3_ population.

Region/hotspot	Gene	SNP	REF allele	ALT allele	W82/MA/OV^1^	Location	SIFT Consequence
F2_GM15:49,385,092-49,442,237	*Glyma.15G261300*	GM15:49,385,259	A	C	A/C/A	3’UTR	N/A
F2_GM15:49,385,092-49,442,237	*Glyma.15G263700*	GM15:49,734,668	A	G	A/G/A	CDS	Missense (Deleterious)
F2_GM15:49,385,092-49,442,237	*Glyma.15G263700*	GM15:49,736,375	T	G	T/G/T	5’UTR	N/A
F2_GM18:1,434,182-1,935,386	*Glyma.18G025600*	GM18:1,893,844	A	G	A/G/A	3’UTR	N/A
F2_GM18:1,434,182-1,935,386	*Glyma.18G025600*	GM18:1,894,078	C	A	C/A/C	3’UTR	N/A

^1^ W82, ‘William 82’; MA, ‘Maple Arrow’; OV, ‘OAC Vision’.

N/A, not available.

### The F2_GM15:49,385,092-49,442,237 hotspot regulates *GmPRR1* and *GmMDE* homologs

**Figure 6:**
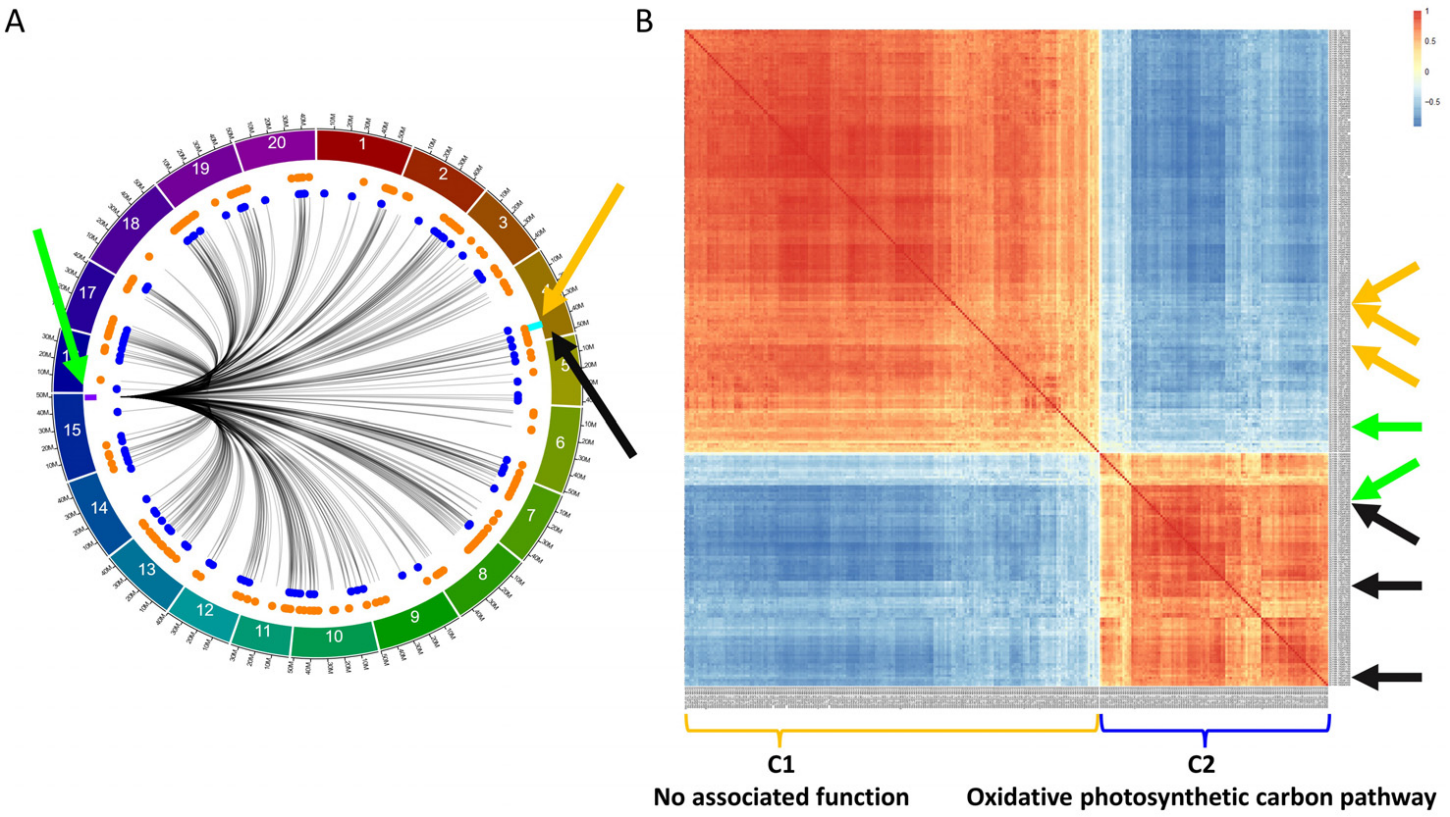
Characterization of the F2_GM15:49,385,092-49,442,237 hotspot and its interaction with *GmPRR1a*, *GmMDE04*, and their homologs in the QS15524_F2:F3_ population. **(A)** Identification of 293 *trans* interactions (285 genes) with the F2_GM15:49,385,092-49,442,237 hotspot. Green arrow, location of the two candidate TFs (*Glyma.15G261300* and *Glyma.15G263700*) and F2_GM15:49,385,092-49,442,237 hotspot (purple rectangle). The locations of *GmPRR1a*, *GmMDE04*, and the *E8-r3* locus are respectively indicated by the orange arrow, black arrow, and light blue rectangle. Orange and royal blue dots respectively represent the genes located in the C1 and C2 clusters. **(B)** Co-expression network with the 285 genes and the two candidate TFs. Orange arrows, location of *GmPRR1a*, and two *PRR* homologs (*GmPRR1d* and *GmARR2*). Black arrows, location of *GmMDE04*, and two *MDE* homologs (*GmMDE13* and *GmMDE19*). Green arrows, location of the candidate TFs. The target genes and candidate TF found in the C1 cluster are indicated with the orange bracket, whereas those found in the C2 cluster are indicated with the royal blue bracket. Based on a functional enrichment analysis, the C2 cluster is associated with the term ‘oxidative photosynthetic carbon pathway’, whereas C1 is not associated with any terms.

In addition to the regulation of *GmLHCA4a*, we also identified several regions regulating two *E8-r3* candidate genes, *GmPRR1a*, previously identified in Gélinas Bélanger et al. (2024a), and *GmMDE04*, proposed by Escamilla et al. (2024). As previously mentioned, both genes were found to be regulated by the F2_GM15:49,385,092-49,442,237 hotspot (**Figure 6A**). To further investigate the networks interacting with this hotspot, we generated a CEN comprising 285 genes based on 293 *trans* interactions (53 from F2_GM15:49,385,092-49,442,075 and 240 from F2_GM15:49,442,075-49,442,237^2^). Along, we used the TF_list_4,611_ to identify candidate TFs and found two, *Glyma.15G261300* and *Glyma.15G263700*, that were located within or close to the region. Although the *Glyma.15G263700* TF was not found within the F2_GM15:49,385,092-49,442,237 hotspot, it was still included in the analysis due to its close proximity with this region (< 300 kbp). Subsequently, we generated a CEN with the 285 target genes along with the 2 candidate TFs (**Figure 6B**) and observed the formation of two separate co-expression clusters: F2_GM15:49,385,092-49,442,237_C1 (184 target genes and one candidate TF, *Glyma.15G261300*) and F2_GM15:49,385,092-49,442,237_C2 (101 target genes and one candidate TF, *Glyma.15G263700*). Although no specific function was found to be significantly associated with the 184 target genes of the F2_GM15:49,385,092-49,442,237_C1 cluster, this cluster contained *GmPRR1a* and two of its homologs (*Glyma.17G102200*/*PRR1d* and *Glyma.07G171200*/*RESPONSE REGULATOR 2*/*ARR2*). In addition, we identified *GmMDE04* and two homologs, *Glyma.13G052100* (*GmMDE13*) and *Glyma.19G034600* (*GmMDE19*), in the F2_GM15:49,385,092-49,442,237_C2 cluster, a group enriched with an oxidative photosynthetic carbon pathway function. Interestingly, one additional *MDE* homolog, *GmMDE17* (*Glyma.17G081200*), was found to be regulated by the F2_GM15:49,385,092-49,442,237 hotspot with ICIM (LOD, 4.75; PVE, 8.56%), but none of the two other software (**Supplementary Table S1L**).

### Functional investigation and variant analysis of the candidate transcription factors regulating *PRR and MDE* homologs

**Figure 7:**
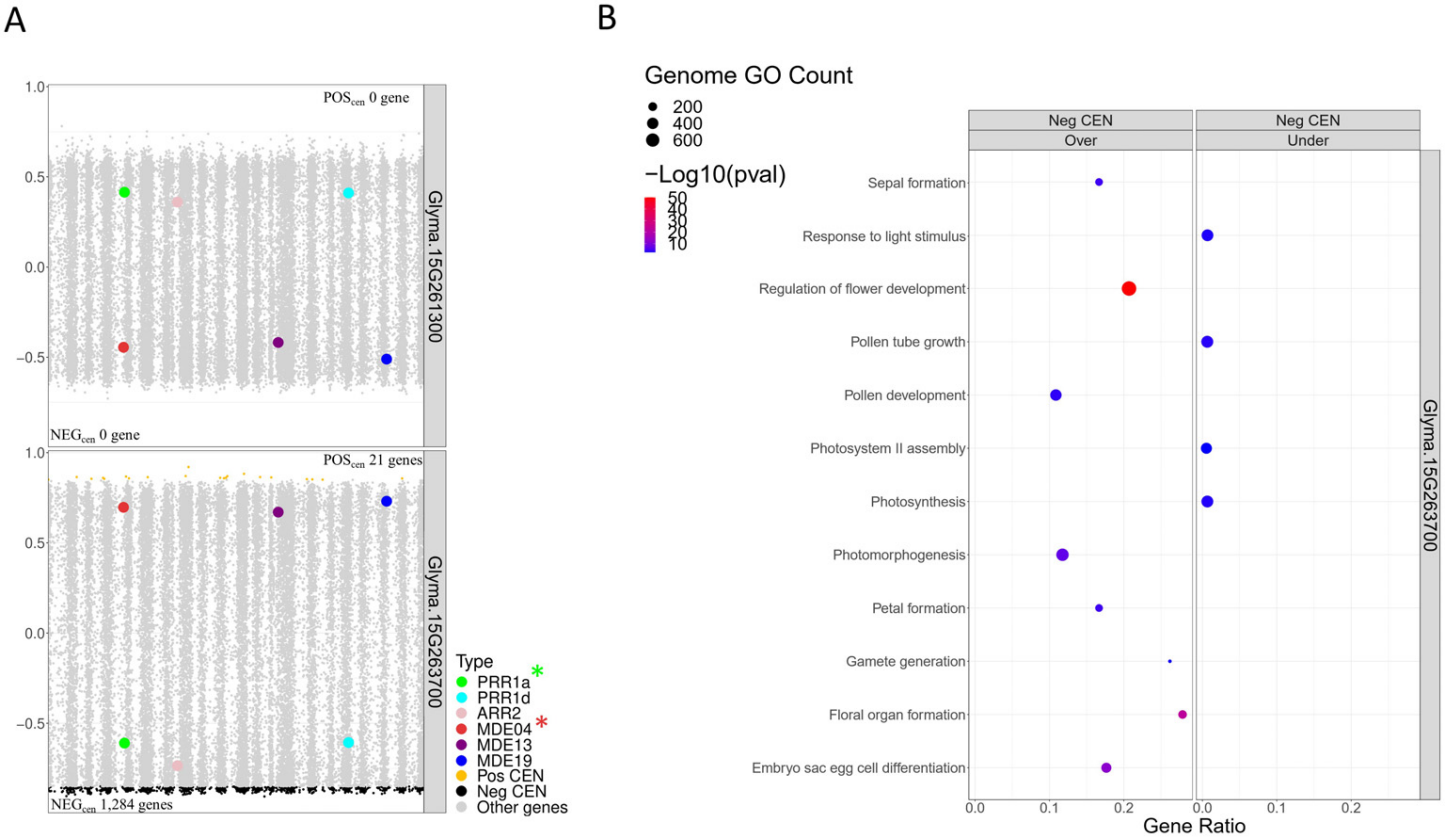
Transcriptome-wide co-expression network for the candidate TFs of the F2_GM15:49,385,092-49,442,237 hotspot. **(A)** Positive and negative TWCENs for the two candidate TFs using PCC thresholds of ≥ 0.85 (POS_TWCEN_) and ≤ -0.85 (NEG_TWCEN_). As shown in the panel, only *Glyma.15G263700* displayed a large NEG_TWCEN_ (1,284 genes). The NEG_TWCEN_ of *Glyma.15G261300* (0 genes) and the POS_TWCEN_ of both candidates (*Glyma.15G263700*, 21 genes; *Glyma.15G261300*, 0 genes) were small. The candidate target genes *GmPRR1a* and *GmMDE04* are highlighted with asterisks. **(B)** Functional annotation of the NEG_TWCEN_ of *Glyma.15G263700*. This NEG_TWCEN_ is strongly enriched with terms associated with flowering and response to light functions. Only gene annotations that are either over-represented (i.e., “Over” facet) or under-represented (i.e., “Under” facet) are displayed in the figure. Non-FRSPD annotations were not displayed for visualization purposes, but are available in **Supplementary Table S1U**.

Following the building of the CEN, we generated TWCENs for both candidate TFs of the F2_GM15:49,385,092-49,442,075 hotspot (**Supplementary Table S1T**). Using a PCC threshold of ≤ -0.85, we found that the NEG_TWCEN_ of *Glyma.15G263700* was large and comprised 1,284 genes, whereas *Glyma.15G261300* had none (**Figure 7A**). Nothing conclusive was found for the POS_TWCEN_ of both candidates as the POS_TWCEN_ of *Glyma.15G263700* comprised only 21 genes and *Glyma.15G261300* had none. Subsequently, we performed a GO enrichment analysis on the NEG_TWCEN_ of *Glyma.15G263700* and discovered that it was significantly enriched with various FRSPD terms associated with flowering, floral organ formation, and photomorphogenesis (**Figure 7B**; **Supplementary Table S1U**). This result is coherent with the fact that the *Glyma.15G263700* candidate TF is annotated with terms related to flower development in Soybase, whereas *Glyma.15G261300* is not annotated with FRSPD functions. To gain insights regarding the putative roles of these two candidate TFs, we investigated their expression profiles, as well as the presence of mutations, in the parental lines. In the QS15524_F2:F3_ parents, *Glyma.15G263700* (Fold change, 2.60; FDR adjusted p-value, 5.15E-04) was differentially expressed, but not Glyma.15G261300 (Fold change, 1.90; FDR adjusted p-value, 0.03) (**Supplementary Figure S2**; **Supplementary Table S1I**). Based on our variant analysis pipeline, we discovered mutations in both genes, with *Glyma.15G263700* displaying a missense mutation predicted to be deleterious by the SIFT algorithm and *Glyma.15G261300* having a 3’UTR variant at position GM15:49,385,259 (**Table 4**).

### *GmPRR1* and *GmMDE* homologs are regulated by the same minor regions

**Figure 8:**
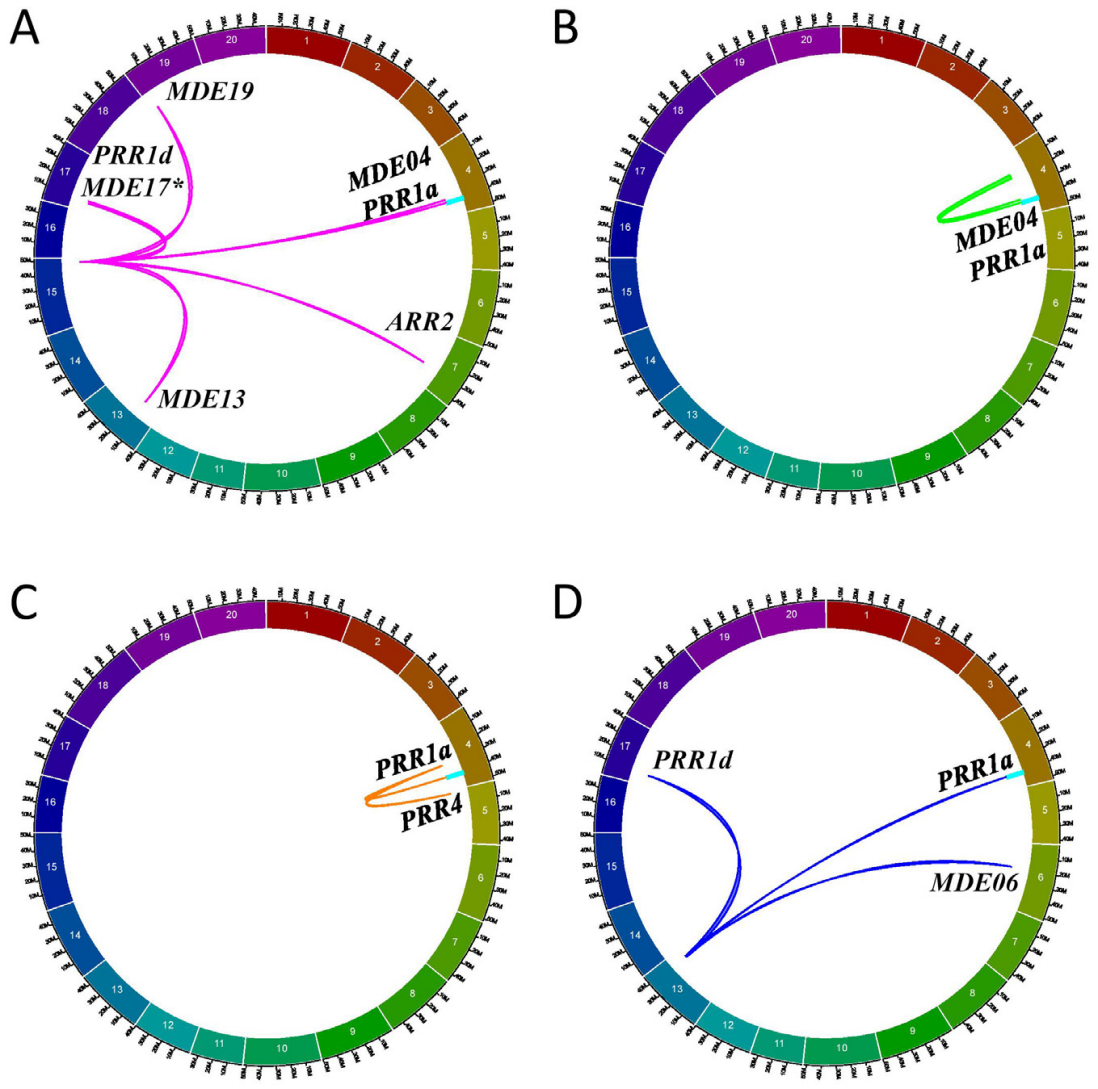
Minor regions regulating the *PRR* and *MDE* homologs in the QS1544_RIL_ population. Circos plots illustrating the interactions between the F2_GM15:49,385,092-49,442,237 **(A)**, RIL_GM04:17,227,512-20,251,662 **(B)**, RIL_GM04:31,389,583-31,525,671 **(C)**, and RIL_GM13:37,289,785-38,620,690 **(D)** regions and the different *PRR* and *MDE* homologs, including the candidate target genes *GmPRR1a* and *GmMDE04* that are located in the *E8-r3* locus (light blue rectangle). The F2_GM15:49,385,092-49,442,237 is regulating an additional *MDE* homolog, *GmMDE17*. The asterisk denotes that this additional homolog has been mapped with only one algorithm (ICIM) instead of two like all the other interactions.

Following these discoveries from the F2_GM15:49,385,092-49,442,237 hotspot (**Figure 8A**), we investigated further to determine whether similar co-regulation events could be observed for minor regions interacting in *trans* with *GmPRR* and *GmMDE* homologs. On the whole, we identified three different minor regions in the QS15544_RIL_ population that were interacting with three *GmPRR* (*GmPRR1a*, *GmPPR1d*, and *Glyma.05G025000/GmPRR4*), and two *GmMDE* (*GmMDE04*, and *Glyma.06G205800*/*GmMDE06*) homologous genes: (i) RIL_GM04:17,227,512-20,251,662 (**Figure 8B**); (ii) RIL_GM04:31,408,946-31,525,671 (**Figure 8C**); and (iv) RIL_GM13:37,289,785-38,620,690 (**Figure 8D**). Interestingly, *GmPPR1d* was found to be regulated by a region located between markers GM04:22,010,259 and GM04:26,441,718 which is adjacent to the minor region RIL_GM04:17,227,512-20,251,662 regulating *GmPRR1a* and *GmMDE04* (**Supplementary Table S1Q**). For each of the regions, the number of candidate TFs ranged between 1 (for RIL_GM04:31,408,946-31,525,671) to 6 (for RIL_GM13:37,289,785-38,620,690) (**Supplementary Table S1V**). We performed the same analyses (i.e. expression analysis, TWCEN, and variant analysis pipeline) for these minor regions than for the F2_GM18:1,434,182-1,935,386 and F2_GM15:49,385,092-49,442,237 hotspots (**Supplementary Table S1V**). Overall, two (out of 11) candidates were annotated with FRSPD terms and none of them were found to be differentially expressed in the parental lines due to FDR-adjusted p-values that were above the threshold. We constructed TWCENs for all of the candidates but only *Glyma.04G135400* was found to have POS_TWCEN_ (446 genes) and NEG_TWCEN_ (445 genes) that were significantly enriched with GO terms including the ‘Phytochrome binding’ term (**Supplementary Table S1U**). Using our custom variant analysis pipeline, we found a total of five mutations in two different candidates (*Glyma.04G135400* and *Glyma.13G285400*) (**Table 5**). Based on these observations, we think that *Glyma.04G135400* is the best candidate for RIL_GM04:17,227,512-20,251,662. Still, we think that more research on neighboring candidate TFs outside of the RIL_GM04:31,408,946-31,525,671 and RIL_GM13:37,289,785-38,620,690 needs to be performed to identify better candidates.

**Table 5:** Single nucleotide polymorphisms for the candidate transcription factors of the QS15544_RIL_ population.

Region/hotspot	Gene	SNP	REF allele	ALT allele	W82/MD/90^1^	Location	SIFT Consequence
RIL_GM04:17,227,512-20,251,662	*Glyma.04G135400*	GM04:19,964,773	A	T	A/T/A	CDS	Missense (Deleterious)
RIL_GM13:37,790,482-38,620,690	*Glyma.13G285400*	GM13:38,627,139	C	G	C/C/G	5’UTR	N/A
RIL_GM13:37,790,482-38,620,690	*Glyma.13G285400*	GM13:38,627,196	A	T	A/A/T	5’UTR	N/A
RIL_GM13:37,790,482-38,620,690	*Glyma.13G285400*	GM13:38,627,262	T	A	T/*/A^2^	5’UTR	N/A
RIL_GM13:37,790,482-38,620,690	*Glyma.13G285400*	GM13:38,627,374	T	A	T/T/A	5’UTR	N/A

^1^ W82, ‘William 82’; MD, ‘AAC Mandor’; 90, ‘9004’.

^2^ An asterisk (*) indicates a heterozygote genotype for that SNP.

N/A, not available.

## Discussion

### The F2_GM18:1,434,182-1,935,386 hotspot is a hub for the coordinated regulation of the light response and photosynthetic mechanisms

Photosystem I (PSI) is located in the thylakoid membrane and is a multiprotein complex that plays a crucial role in oxygenic photosynthesis by oxidizing plastocyanin and reducing ferredoxin (Sláma et al., 2023). PSI is divided into the core complex and the outer antenna complexes (also known as Light-Harvesting Complex I; LHCI). The role of the LHCI is to harvest light and transfer the excitation energy of the electrons to the reaction center. The antenna of the Light-Harvesting Complex I comprises four subunits which are the products of *Lhca1-4* genes in *Arabidopsis*. We previously demonstrated that the *E8-r3* region (GM04:41,808,599-42,376,237) regulates the number of days to maturity under a constant short-day photoperiod in both QS15524_F2:F3_ and QS15544_RIL_. Based on these observations, we proposed the *Glyma.04G167900* (*GmLHCA4a*) gene as a potential candidate for this region using a candidate SNP analysis (Gélinas Bélanger et al., 2024a). In previous studies, *GmLHCA4a* has been identified as a candidate for the *q4–2* locus regulating leaf-related traits (i.e. leaf size and shape) and chlorophyll content (Yu et al., 2020). Furthermore, *GmLHCA4a* has been suggested to be involved in the number of days to flowering as Liu et al. (2021) observed a 1.8-day difference between two *GmLHCA4a* haplotypes under short-day growth conditions. In the present study, we demonstrated that the F2_GM18:1,434,182-1,935,386 hotspot regulates the C1 cluster, a group of 79 genes regulating photosynthesis and light response mechanism in the QS15524_F2:F3_ population. The F2_GM18:1,434,182-1,935,386_C1 cluster includes six Light-Harvesting Complex homologs and several genes associated with PSI (e.g. *Glyma.10G042100*/*PHOTOSYSTEM I SUBUNIT E-2*) and PSII (e.g. *Glyma.10G089300*, *Glyma.10G089500* and *Glyma.15G275600*; all *PHOTOSYSTEM II 5 KDA* proteins). In soybean, 62 proteins, including 34 *LHC A/B* proteins, have been predicted to be involved in the regulation of the Light-Harvesting Complex (Lan et al., 2022). Consequently, the genes that were identified for the light response subcluster represent only a fraction of the LHC genes within the soybean genome.

### The *Glyma.18G025600* gene is the best candidate regulator for the *LHC* homologs

From these observations, we identified three candidate TFs (*Glyma.18G020900*, *Glyma.18G025600*, and *Glyma.18G025800*) located within the F2_GM18:1,434,182-1,935,386 hotspot. Based on our co-expression analysis, *Glyma.18G020900* and *Glyma.18G025600* were strongly co-expressed with the *LHC* homologs, but only *Glyma.18G025600* had POS_TWCEN_ and NEG_TWCEN_ associated with photosynthetic and photosystem I/II regulation functions. Interestingly, *Glyma.18G025600* harbored two mutations in its 3’UTR in OV, whereas none were found in *Glyma.18G020900*. To the best of our knowledge, this is the first time *Glyma.18G025600* is proposed as a candidate for the transcriptional regulation of these six *LHC* homologs and more largely to the targets of the F2_GM18:1,434,182-1,935,386_C1 cluster in soybean. The *LOB DOMAIN-CONTAINING PROTEIN 21* gene (*Glyma.18G025600*; *GmLBD21*) is the ortholog of AT3G11090 (*AtASL12*/*AtLBD21*) in Arabidopsis which belongs to the class 1a of the AS2 protein family (Iwakawa et al., 2002; Shuai et al., 2002). The *AtAS2* gene (AT1G65620) encodes a domain that includes a leucine-zipper-like sequence in its amino-terminal half and a cysteine repeat (Matsumura et al., 2009). On a functional level, *AtAS2* plays a role in the expansion of flat leaf lamina in Arabidopsis as *AtAS2* overexpressing and loss-of-function Arabidopsis mutants respectively exhibited upwardly and downwardly curled leaves (Iwakawa et al., 2002). The gene *DOWN IN DARK AND AUXIN1* (AT3G27650; *AtDDA1/AtLBD25*/*AtASL3*), a gene closely associated with *LATERAL ORGAN BOUNDARIES* (*AtLOB*) and *ASYMMETRIC LEAVES2* (*AS2*), has been suggested to be implicated in photomorphogenesis and auxin response as *dda1-1* plants display aberrant hypocotyl elongation and reduced sensitivity to auxin phenotypes (Mangeon et al., 2011). Overall, the current pieces of evidence suggest that *Glyma.18G025600* is the best candidate for the F2_GM18:1,434,182-1,935,386 hotspot.

### *GmPRR1a* and *GmMDE04* are co-regulated by the same regions

The *GmPRR1a* and *GmMDE04* genes and their homologs are known to have critical impacts on photoperiodic flowering in *Arabidopsis* and soybean. The soybean PSEUDO RESPONSE REGULATORs 1a and 1d are orthologs of the *Arabidopsis* DNA-binding transcription factor TIMING OF CAB EXPRESSION 1/PSEUDO-RESPONSE REGULATOR 1 (AtTOC1/AtPRR1) which contains a CCT (CONSTANS, CO-like, TOC1) domain in the C terminus and a pseudo receiver domain in the N terminus (Gendron et al., 2012). In *Arabidopsis*, this protein is known to be involved in the phytochrome regulation of circadian gene expression and photomorphogenic response (Más et al., 2003) and thus acts as a molecular bridge between environmental cues and clock outputs. In soybean, *GmMDE04* (also named *GmFULb*) is involved in the *E1*-*GmMDEs*-*GmFT2a/5a*-*Dt1* signaling pathway and responds to photoperiod at the transcript level (Zhai et al., 2022). Overexpression experiments have demonstrated that the *GmMDE06* homolog acts downstream of *E1* in the induction of the flowering process, increases the expression of *GmFT2a/GmFT5a* and promotes the termination of stem growth by repressing *Dt1* (Zhai et al., 2022). According to Zhai et al. (2022), *GmMDE04* is significantly expressed under short-day conditions versus long-day conditions in the ‘Harosoy-E1’, ‘Zhonghuang 13’ and ‘Gaofeng1’ backgrounds but not in ‘Harosoy-e1’, ‘Kariyutaka’, and ‘Sidou 11’. This gene is the ortholog of the *Arabidopsis* gene *AGAMOUS-LIKE 8*/*FRUITFUL* which induces global proliferative arrest (i.e. the coordinated arrest of all active meristems) by repressing members of the *APETALA2* (*AtAP2*) clade involved in the maintenance of the shoot apical meristem (Martínez-Fernández et al., 2020). As a whole, the suppression in *E1* expression has been demonstrated to be tightly associated with the photoperiod-insensitive expression of *GmMDE*s (Zhai et al., 2022). Structurally, *GmMDE04* and *GmMDE06* exhibit a higher degree of similitude between each other than for the five other *MDE* genes (results not shown).

In the present study, we identified two co-regulation events between *GmPRR1a* and *GmMDE04*. The first was found in QS15524_F2:F3_ (F2_GM15:49,385,092-49,442,237 hotspot), whereas the second was discovered in QS15544_RIL_ (RIL_GM04:17,227,512-20,251,662). In addition, we also identified two other co-regulation events between *PRR* (i.e., *GmPRR1d*, *GmARR2*, and *GmPRR4*) and *MDE* (*GmMDE06*, *GmMDE13*, *GmMDE17*, and *GmMDE19*) homologs in the QS15544_RIL_ population. Each of these regulation events were identified by at least two algorithms, except the interaction between *GmMDE17* and the F2_GM15:49,385,092-49,442,237 hotspot, thus indicating the robustness and reliability of these interactions. Still, we consider the interaction between *GmMDE17* and the F2_GM15:49,385,092-49,442,237 hotspot to be robust as we consider ICIM to be the one of the most reliable algorithms currently available to researchers.Regarding RIL_GM04:17,227,512-20,251,662, we discovered that this locus is located near the *E8-r1* locus (RIL_GM04:16,974,874-17,152,230), a locus discovered in the same study as for *E8-r3* and in the same population (Gélinas Bélanger et al., 2024a); however, this locus was found for the pod-filling trait under field conditions and was not considered for the present study. Still, the data generated in Gélinas Bélanger et al. (2024a) demonstrate that a critical regulator is found within the same genomic region.

For the co-regulation events associated with the F2_GM15:49,385,092-49,442,237 hotspot, two genes (*Glyma.15G261300* and *Glyma.15G263700*) have been proposed as candidate regulators. At present, several lines of evidence (i.e. functional annotations, TWCEN functions, type of prevailing mutations, and more) suggest that *Glyma.15G263700* (*ALTERED PHLOEM DEVELOPMENT*/*GmAPL*; also called *GmFE*) is the best candidate. The *GmAPL* is the ortholog of AT1G79430 in *Arabidopsis*, a phloem-specific Myb-related protein involved in the photoperiodic induction of flowering (Abe et al., 2015). Abe et al. (2015) have demonstrated that a missense mutation causing a glycine (G) to glutamic acid (E) substitution causes a late-flowering phenotype in *Atfe* mutants. Using expression analysis, Abe et al. (2015) have shown that a fully functioning *AtFE* allele is required for the transcriptional activation of *FLOWERING LOCUS T INTERACTING PROTEIN 1* (*AtFTIP1*), a critical gene involved in the selective trafficking of *AtFT* protein from phloem companion cells to sieve elements (Liu et al., 2012).

## Conclusion

We developed a novel eQTL mapping pipeline that enabled us to identify hundreds of transient *cis* and *trans* interactions in the QS15524_F2:F3_ and QS15544_RIL_ soybean populations. From the *trans* interactions, we identified four hotspots involved in the regulation of FRSPD functions: (i) F2_GM06:39,892,719-43,437,125, F2_GM17:5,431,473-7,260,313 and F2_GM18:1,434,182-1,935,386 in QS15524_F2:F3_; and (ii) the RIL_GM04:10,812,813-10,985,437 in QS15544_RIL_. Deeper investigations identified *trans* regulatory events between: (i) F2_GM18:1,434,182-1,935,386 and *GmLHCA4a*; and (ii) several regions identified in QS15524_F2:F3_ and QS15544_RIL_ and two candidate genes (*GmPRR1a* and *GmMDE04*) along with some homologs (*GmPRR1d*, *GmPRR4*, and *GmMDE06*). Using an approach combining the analysis of predicted TFs, TWCEN, annotated functions, and genomic variants, we identified several candidates for these regions of interest, with a focus on *GmLBD21* (*Glyma.18G025600*) and *GmAPL* (*Glyma.15G263700*). Overall, the discoveries regarding the loci regulating the three candidate genes for the *E8-r3* region (*GmLHCA4a*, *GmPRR1a*, and *GmMDE04*) represent only a small proportion of the *trans* and *cis* interactions captured with our combinatorial mapping approach. These findings demonstrate the potential of eQTL interactions and hotspot mapping combined with co-expression analyses to identify a large number of TF-related regulatory events and narrow the number of potential TF candidates.

## Data Availability

The datasets presented in this study can be found in online repositories. The names of the repository/repositories and accession number(s) can be found below: https://www.ncbi.nlm.nih.gov/, BioProject PRJNA1035514.

## Footnotes

1 One gene was found to be regulated by both regions.

2 Four genes were found to be regulated by both regions.

## Supplementary material

Supplementary Figure 1 Differentially expressed candidate genes for the *E8-r3* locus in the QS15524_F2:F3_ and QS15544_RIL_ parental lines. Heatmaps showing the number of DEGs in the QS15524_F2:F3_ **(A)** and QS15544_RIL_ **(C)** parental lines. Volcano plots showing the differentially expressed candidate genes in the QS15524_F2:F3_ **(B)** and QS15544_RIL_ **(D)** parental lines. Two candidate *E8-r3* genes (*GmCDF3* and *GmMDE04*) have been found to be upregulated in the QS15524_F2:F3_ parents, whereas none of the four candidates were found to be differentially expressed in the QS15544_RIL_ parents.

Supplementary Figure 2 Differentially expressed candidate transcription factors in the QS15524_F2:F3_ parents. Three candidate TFs (*Glyma.15G263700*, *Glyma.18G020900*, and *Glyma.18G025800*), have been found to be differentially expressed in the QS15524_F2:F3_ parental lines.
