# Supplementary figures and images for: Integrated eQTL mapping approach reveals genomic regions regulating candidate genes of the E8-r3 locus in soybean
Source: Front Plant Sci. 2024 Nov 12;15:1463300. doi: 10.3389/fpls.2024.1463300 (PMC11589821; doi:10.3389/fpls.2024.1463300)

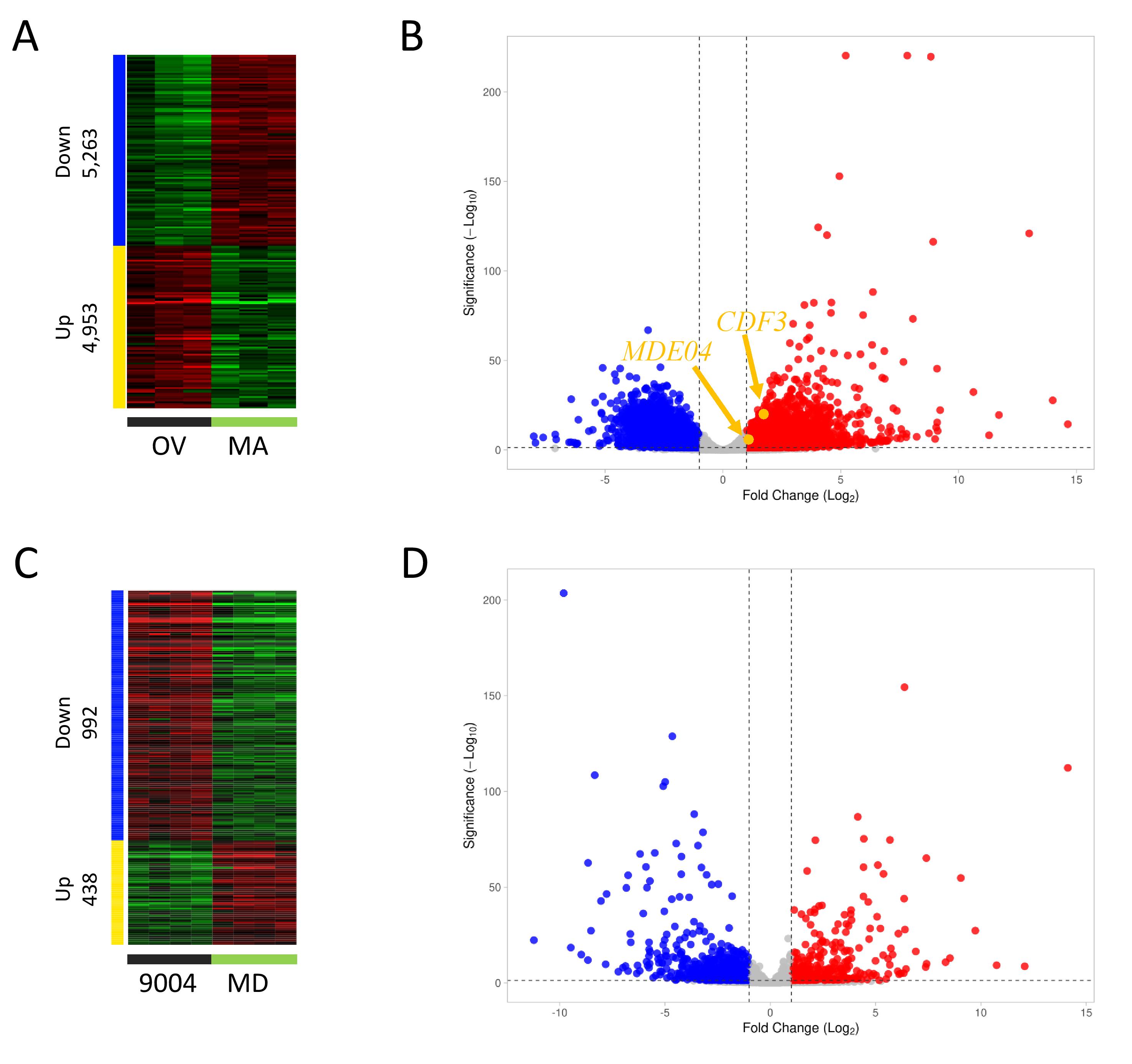

Supplement: Supplementary Figure 1 — Differentially expressed candidate genes for the E8-r3 locus in the QS15524F2:F3 and QS15544RIL parental lines. Heatmaps showing the number of DEGs in the QS15524F2:F3 (A) and QS15544RIL (C) parental lines. Volcano plots showing the differentially expressed candidate genes in the QS15524F2:F3 (B) and QS15544RIL (D) parental lines. Two candidate E8-r3 genes (GmCDF3 and GmMDE04) have been found to be upregulated in the QS15524F2:F3 parents, whereas none of the four candidates were found to be differentially expressed in the QS15544RIL parents. [file Image1.jpeg]

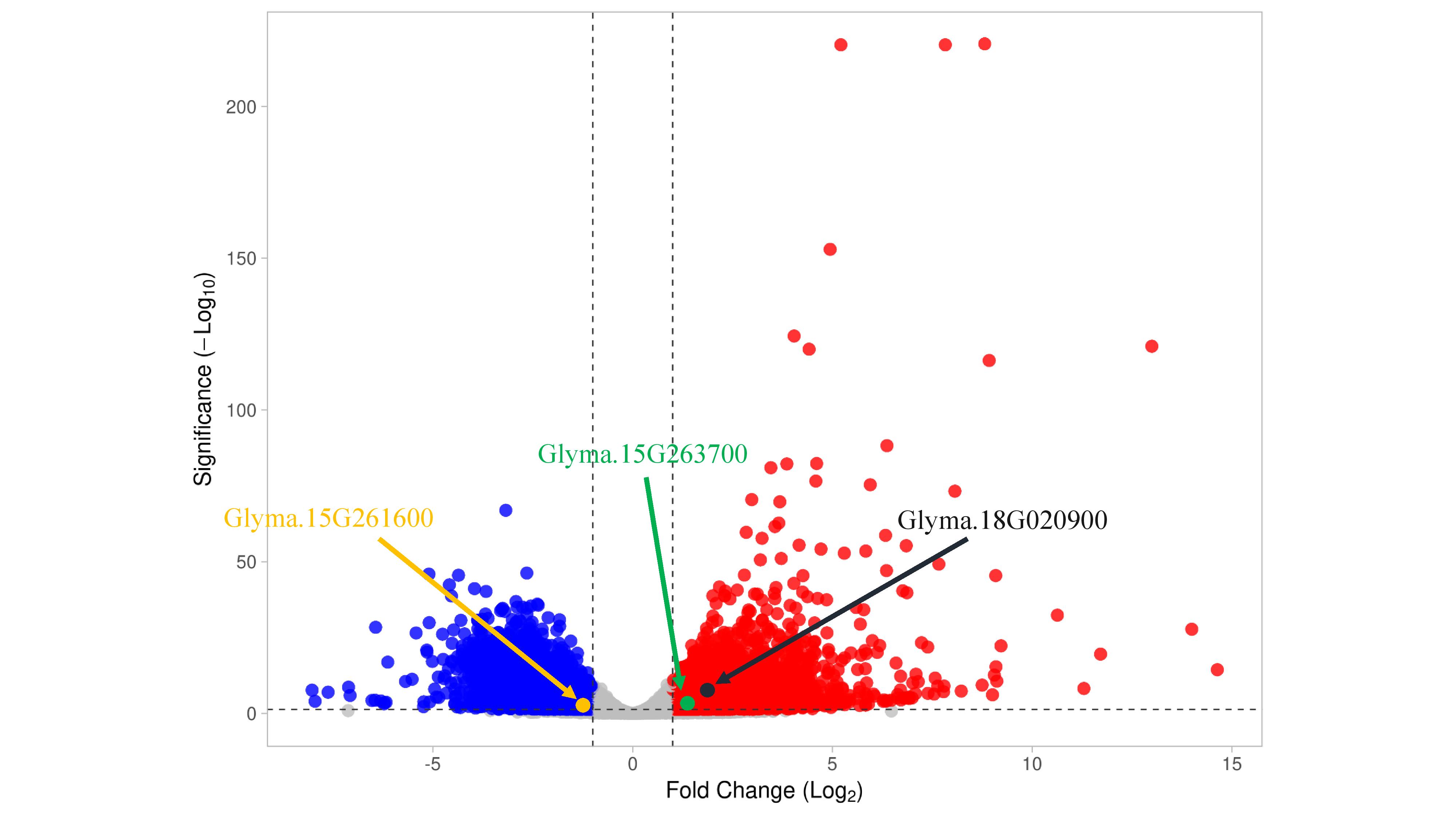

Supplement: Supplementary Figure 2 — Differentially expressed candidate transcription factors in the QS15524F2:F3 parents. Three candidate TFs (Glyma.15G263700, Glyma.18G020900, and Glyma.18G025800), have been found to be differentially expressed in the QS15524F2:F3 parental lines. [file Image2.jpeg]
